# PD-1/PD-L1 inhibitors plus carboplatin and paclitaxel compared with carboplatin and paclitaxel in primary advanced or recurrent endometrial cancer: a systematic review and meta-analysis of randomized clinical trials

**DOI:** 10.1186/s12885-023-11654-z

**Published:** 2023-11-29

**Authors:** Francisco Cezar Aquino de Moraes, Eric Pasqualotto, Lucca Moreira Lopes, Maria Eduarda Cavalcanti Souza, Anna Luíza Soares de Oliveira Rodrigues, Artur Menegaz de Almeida, Carlos Stecca, Marianne Rodrigues Fernandes, Ney Pereira Carneiro dos Santos

**Affiliations:** 1 Oncology Research Center, Federal University of Pará, University Hospital João de Barros de Barreto, Rua dos Mundurucus, nº4487, Belém, 66073-000 PA Brazil; 2 Federal University of Santa Catarina, Florianópolis, 88040-900 Santa Catarina Brazil; 3 Sciences Medical School of Santos, Santos, São Paulo, 11045-101 Brazil; 4 University of Pernambuco, Recife, 50670-901 Pernambuco Brazil; 5 University Center of João Pessoa, João Pessoa, Paraíba, 58053-000 Brazil; 6 Federal University of Mato Grosso, Sinop, 78550-704 Mato Grosso Brazil; 7 Mackenzie Evangelical University Hospital, Curitiba, 80730-150 Paraná Brazil

**Keywords:** Endometrial cancer, Immune Checkpoint inhibitors, Chemotherapy, Mismatch repair–deficient

## Abstract

**Background:**

Paclitaxel and carboplatin is the standard chemotherapy for the treatment of advanced or recurrent endometrial cancer. However, the benefit of adding programmed cell death 1 (PD-1)/programmed death ligand 1 (PD-L1) inhibitors to chemotherapy is still unclear.

**Method:**

We searched PubMed, Scopus, Cochrane, and Web of Science databases for randomized controlled trials that investigated PD-1/PD-L1 inhibitors plus carboplatin and paclitaxel compared with carboplatin and paclitaxel in primary advanced or recurrent endometrial cancer. We computed hazard ratios (HRs) or risk ratios (RRs) for binary endpoints, with 95% confidence intervals (CIs). We used DerSimonian and Laird random-effect models for all endpoints. Heterogeneity was assessed using I^2^ statistics. R, version 4.2.3, was used for statistical analyses.

**Results:**

A total of three studies and 1,431 patients were included. Compared with carboplatin plus paclitaxel-based chemotherapy, progression-free survival (PFS) rate (HR 0.32; 95% CI 0.23–0.44; *p* < 0.001) and overall survival (OS) at 30 months (RR 3.13; 95% CI 1.26–7.78; *p* = 0.01) were significant in favor of the PD-1/PD-L1 inhibitors plus carboplatin and paclitaxel group in the mismatch repair–deficient subgroup. However, there were no significant differences in the mismatch repair–proficient subgroup for PFS (HR 0.74; 95% CI 0.50–1.08; *p* = 0.117) or OS at 30 months (RR 2.24; 95% CI 0.79–6.39; *p* = 0.13).

**Conclusion:**

Immunotherapy plus carboplatin-paclitaxel increased significantly PFS and OS among patients with advanced or recurrent endometrial cancer, with a significant benefit in the mismatch repair–deficient and high microsatellite instability population.

**Supplementary Information:**

The online version contains supplementary material available at 10.1186/s12885-023-11654-z.

## Background

Endometrial cancer is currently the sixth most common cancer among women worldwide, and is expected to be the fourth leading cause of death among female tumors by 2040 [1–4]. Carboplatin plus paclitaxel is the standard chemotherapy for first-line treatment for advanced or primary recurrent endometrial cancer, however, outcomes still remain dismal, with less than 3 years of median overall survival [5–7].

In endometrial cancer, mismatch repair deficiency (dMMR) and high microsatellite instability (MSI-H) are present in about 25–30% of cases [8, 9]. The high expression of the programmed cell death 1 (PD-1) receptor and its ligands, programmed death ligand 1 (PD-L1) and programmed death ligand 2 (PD-L2), associated with the high mutational load of endometrial cancer dMMR/MSI-H make this subtype more sensitive to Immune Checkpoint Inhibitors (ICIs), particularly anti-PD-1 and anti-PD-L1 agents [10–12].

The combination of Pembrolizumab (PD-1 inhibitor) and Lenvatinib (tyrosine kinase inhibitor) is approved by the Food and Drug Administration (FDA) for the treatment of mismatch repair–proficient (pMMR) endometrial cancer who have relapsed to at least one line of cytotoxic chemotherapy [13–15]. The hypothesis that the combination of ICIs and chemotherapy may benefit patients with advanced or recurrent endometrial cancer is well-founded. This is based on several factors, including increased tumor antigenic diversity resulting from genetic mutations acquired during clonal evolution. These mutations could synergistically interact with the immunogenic effects of cytotoxic chemotherapy, leading to increased levels of cytotoxic T lymphocytes (TCD8+) in comparison to regulatory T cells (T-reg). Moreover, this combination treatment might enhance dendritic cell (DC) activation by inhibiting the STAT6 pathway, as well as foster antigen cross-presentation and inhibition of myeloid lineage-derived suppressor cells. These factors collectively create a conducive environment for a positive response to treatment [16–19].

Therefore, in this systematic review and meta-analysis of randomized clinical trials (RCTs), our aim is to investigate and clarify the potential benefits in terms of PFS, OS, and safety when utilizing PD-1/PD-L1 inhibitors in combination with carboplatin and paclitaxel chemotherapy, as compared to using carboplatin plus paclitaxel chemotherapy alone, in patients with advanced or recurrent endometrial cancer.

## Methods

### Protocol and registration

This systematic review followed the Preferred Reporting Items for Systematic Reviews and Meta-Analysis (PRISMA) guidelines [20]. The protocol was registered in the International Prospective Register of Systematic Reviews (PROSPERO) with registration number CRD42023445890.

### Eligibility criteria

Studies that met the following eligibility criteria were included: (1) RCTs; (2) carboplatin AUC (area under the plasma or serum concentration-time curve) 5 mg/mL and paclitaxel (175 mg/m^2^) chemotherapy-based with or without PD-1/PD-L1 inhibitors; (3) patients ≥ 18 years of age with advanced, recurrent, or metastatic primary endometrial cancer that was not amenable to curative therapy; (4) patients with Eastern Cooperative Oncology Group (ECOG) performance status score of 0, 1, or 2; (5) patients with stage III or IV disease (International Federation of Gynecology and Obstetrics [FIGO]) according to the Response Evaluation Criteria for Solid Tumors (RECIST), version 1.1; or recurrent disease without prior treatment with systemic therapy; or previously treated with neoadjuvant or adjuvant therapy and had relapse or progression for at least 6 months after completion of treatment (first recurrence); and (6) patients could have had prior radiotherapy or hormone therapy [21]. We excluded studies with overlapping populations, non-randomized clinical trials and studies with no outcomes of interest. Inclusion and exclusion criteria for the RCTs included in the systematic review and meta-analysis are detailed in Table S1.

Thus, we sought to answer the following question: How effective is the addition of PD-1/PD-L1 inhibitors to carboplatin and paclitaxel vs. carboplatin and paclitaxel for first-line treatment of advanced or recurrent endometrial cancer?

### Search strategy

Pubmed, Cochrane Library, Scopus, and Web of Science were systematically searched on July 07, 2023. The search strategy with the MeSH terms is detailed in Table S2, Supplementary Material.

Aiming the inclusion of additional studies, the references of the included articles and systematic reviews of the literature were evaluated and an alert was established for notifications in each database, in case a study corresponding to the consultation carried out was eventually published. Those found in the databases and in the references of the articles were incorporated into the reference management software (EndNote®, version X7, Thomson Reuters, Philadelphia, USA). Duplicate articles were automatically and manually excluded. Titles and abstracts of articles found in the databases were analyzed independently by two reviewers (L.M.L. and A.M.A.). Disagreements were resolved by consensus between the two authors and the senior author (L.M.L., A.M.A. and N.P.C.S).

### Data extraction

The following baseline characteristics were extracted: (1) ClinicalTrials.gov Identifier; (2) study design; (3) regimen details in experimental and control arm; (4) number of patients allocated for each arm; and (5) main patient’s characteristics.

The ensuing outcomes of interest were extracted: (1) PFS, defined as the time from patient randomization to disease progression or death from any cause; (2) OS, defined as the period of time, from the start of treatment, that patients are still alive; and (3) adverse events, defined as an unwanted effect of a treatment, which were evaluated by the Common Terminology Criteria for Adverse Events, version 5.0, in the included RCTs [22]. Two authors (A.L.S.O.R and M.E.C.S) collected pre-specified baseline characteristics and outcome data.

Where available, the full protocol of each study was consulted to verify study objectives, population, and other relevant information regarding study design and conduction. For publications reporting results from the same study, the most recent or complete publication reporting the information of interest was considered.

### Endpoints and subgroup analysis

Outcomes of interest included: (1) PFS; (2) OS; patients with any grade of (3) fatigue; (4) peripheral sensory neuropathy; (5) nausea; (6) constipation; (7) diarrhea; (8) dyspnea; (9) rash; (10) anemia; (11) arthralgia; (12) neutropenia or neutrophil count decreased; patients with grade ≥ 3 of (13) anemia; (14) dyspnea; and (15) neutropenia or neutrophil count decreased.

We performed a subgroup analysis for patients with MMR or pMMR to assess PFS and OS.

### Risk of bias assessment

The Cochrane Collaboration tool for assessing risk of bias in randomized trials (RoB 2) was utilized for quality assessment of individual randomized studies [23]. Three authors (E.P., L.M.L. and F.C.A.M.) independently conducted the risk of bias assessment and disagreements were resolved by consensus. Each trial was assigned a score of high, low, or unclear risk of bias across five domains: randomization process, deviations from intended interventions, missing outcomes, measurement of outcomes, and selection of reported results. Funnel-plot analyses were employed to examine publication bias [24].

### Statistical analysis

Hazard ratio (HR) was used to analyze the PFS. We consider HR > 1 favoring the control group and HR < 1 favoring the intervention group. Those evaluated with binary outcomes were assessed with risk-ratios (RRs), with 95% confidence intervals (CIs). The Cochrane *Q*-test and I^2^ statistics were used to assess heterogeneity; *P* values > 0.10 and I^2^ values > 25% were considered to indicate significance for heterogeneity [25]. The Sidik-Jonkman estimator was used to calculate the tau^2^ variance between studies [26]. We used DerSimonian and Laird random-effect models for all endpoints [27]. Publication bias was explored using Egger’s linear regression test [28]. The packages used were “meta” and “metagen”. Statistical analyses were performed using R statistical software, version 4.2.3 (R Foundation for Statistical Computing).

## Results

### Search results and characteristics of included studies

**Fig. 1:**
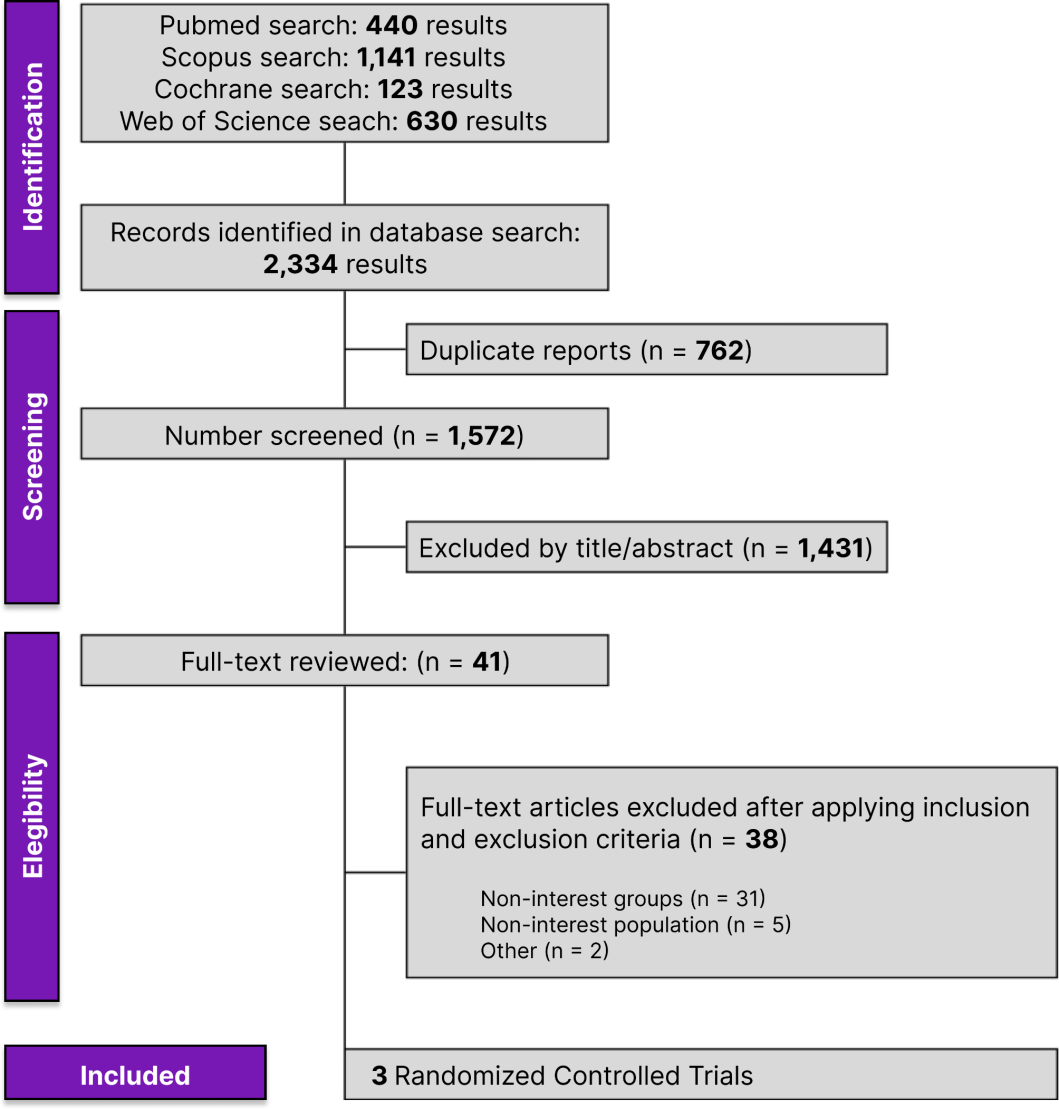
PRISMA flow diagram of study screening and selection

The selection was detailed in a PRISMA flow diagram (Fig. 1). A total of 2,334 references were retrieved in our systematic search. After the removal of duplicate records, and the assessment of the studies based on title and abstract, 2,193 references were excluded and 41 full-text manuscripts were eligible and thoroughly reviewed for inclusion and exclusion criteria. Of these, three clinical trials in 41 references satisfied the eligibility criteria and formed the scope of the analysis, comprising 1,431 patients [29–31].

A total of 713 patients with endometrial cancer were randomized to PD-1/PD-L1 plus carboplatin-paclitaxel and 718 patients to carboplatin-paclitaxel chemotherapy. Most of the patients had mismatch repair–proficient (n = 1018, 71.14%); 400 (27.95%) had mismatch repair–deficient, microsatellite instability–high tumors.

Study patient baseline characteristics of the included studies are summarized in Table 1. In the overall population, 664 of 1431 patients had received a previous anticancer therapy: 468 (30.70%) were treated with radiotherapy, and 196 (13.70%) received chemotherapy. Regarding the histological diagnosis, 844 (58.98%) patients had endometrioid subtype, 276 (19.29%) serous, 83 (5.8%) adenocarcinoma, 59 (4.12%) clear cell, 45 (3.14%) mixed epithelial, 31 (2.17%) dedifferentiated, and 10 (0.7%) pending types of tumors.

**Table 1:** Design and Characterístics of Studies Included in the Meta-analysis

Study	Design/NCT	Folow-up^†^	Age, yr ^†^	Race - no.(%)	ECOG - no (%)	Histologic - no (%)	MMR status - no (%)	Previous therapy no (%)	Treatment^§^
MITO END-3, 2023	RCT - Phase II/ NCT03503786	23·3 months (13.2–29.6)	I: 66 C: 65	NA	I: 0–49 (77.78) 1–14 (22.22) C:0–52 (83.87) 1–10 (16.13)	I:Endometriod 44 (69.84) Serous − 10 (15.87) Others*-8 (12.7) C:Endometriod − 46 (74.19) Serous − 9 (14.52) Others*- 7 (11.29)	I: dMMR − 26 (41.27) pMMR − 35 (55.56) C: dMMR − 31 (50) pMMR-19 (30.65)	I: AC: 20 (32) AR: 28 (45): C: AC: 14 (22) AR: 29 (46)	Avelumab 10 mg
NRG-GY018, 2023	RCT - Phase III/ NCT03914612	12- dMMR 7.9 - pMMR	I: 66 C: 65.1	I: White − 189 (77.14) Black − 28 (11.43) Other** − 27 (11.02) C: White − 191 (76.71) Black − 31 (12.45) Other** − 27 (10.84)	I: 0–268(66.17) I − 127 (31.36) II − 10 (2.47) C: 0–271 (66.42) I − 123 (30.15) II − 14 (3.43)	I:Endometriod − 246 (60.74) Serous − 82 (20.25) Others*- 77 (19.01) C:Endometriod − 238 (58.33) Serous − 73 (17.89) Others*- 97 (23.7)	I: dMMR- 112 (27.65) pMMR − 293 (72.35) C: dMMR − 113 (27.70) pMMR- 295 (72.30)	I: AC: 77 (19.01) AR: 155 (38.27) C: AC: 85 (20.83) AR: 174 (42.65)	Pembrolizumab 200 mg
RUBY, 2023	RCT - Phase III/ NCT03981796	24.8 ( 19.2 to 36.9)	I: 64 C: 65	I: White − 304 (75.06) Black − 56 (13.83) Other** − 68 (16.79) C: White − 298 (73.04) Black − 60 (14.71) Other** − 65 (15.93)	I:0–145(60.16) 1–96 (39.83) C: 0–160 (65.04) 1–86 (34.95)	I:Endometriod − 134 (54.69) Serous 50 (20.41) Others*- 19 (7.76) C:Endometriod − 136 (54.62) Serous − 52 (20.88) Others*- 20 (8.02)	I: dMMR: 53 (21.63) pMMR:192 (78.36) C: dMMR: 65 (26.10) pMMR: 184 (73.89)	I: AR: 41 (16.73) C: AR: 41 (18.07)	Dostarlimab 500 mg

* Others: Dedifferentiates or unidifferentieates, Mixed epithelial, Pending, Adenocarcinoma, Clear cell; ** Others: American Indian or native, Unknown, Latin or hispanic, Caribeean, Asian, Multiracial; † Median range; § Day 1, IV; AC, Adjuvant chemotherapy; AR, Adjuvant radiotherapy; ECOG, Eastern Cooperative Oncology Group; dMMR, mismatch repair–deficient; MMR, Mismatch repair; NA, not available; pMMR, mismatch repair–proficient

### Results based on outcome

#### Progression-free-survival

**Fig. 2:**
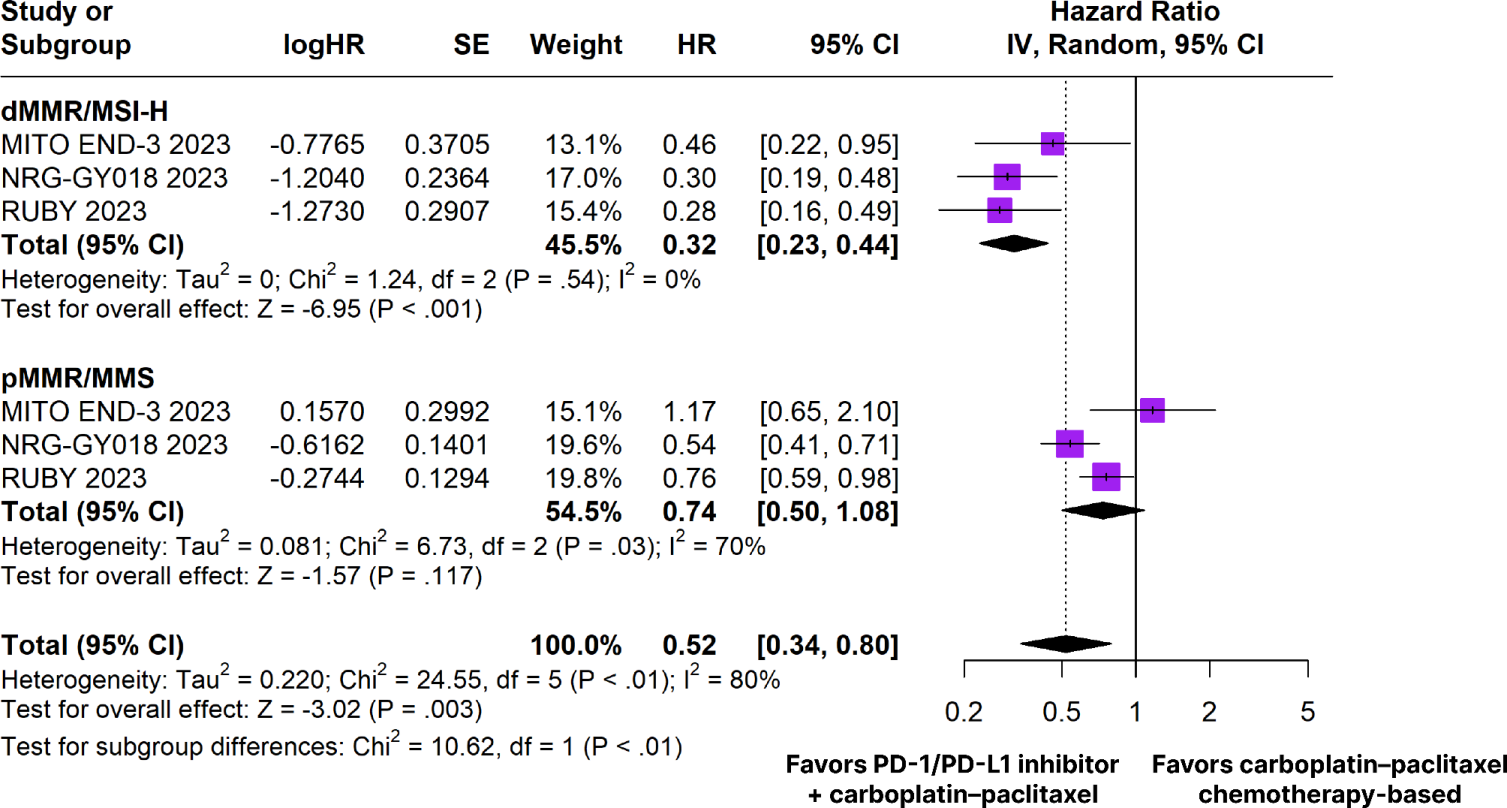
Progression-free survival of patients with endometrial cancer treated with PD-1/PD-L1 inhibitors plus carboplatin and paclitaxel chemotherapy-based versus carboplatin plus paclitaxel chemotherapy-based. dMMR/MSI-H, mismatch repair–deficient/microsatellite instability; pMMR/MMS, mismatch repair–proficient/mismatch repair

All three RCTs analyzed PFS outcome. Among the patients with endometrial cancer, the estimated PFS rate was significantly in favor of the PD-1/PD-L1 inhibitors plus carboplatin and paclitaxel chemotherapy-based group (HR 0.52; 95% CI 0.34–0.80; *p* < 0.01; I²=80%; Fig. 2). In the dMMR subgroup, the estimated PFS rate was significantly in favor of the PD-1/PD-L1 inhibitors plus carboplatin and paclitaxel chemotherapy-based group (HR 0.32; 95% CI 0.23–0.44; *p* < 0.001; I²=0%; Fig. 2). However, there was no significant difference between groups in the pMMR subgroup (HR 0.74; 95% CI 0.50–1.08; *p* = 0.117; I²=70%; Fig. 2).

In patients with dMMR tumors, there was a significant difference from baseline in favor of the PD-1/PD-L1 inhibitors plus carboplatin and paclitaxel chemotherapy-based group in PFS at 6 months (RR 1.32; 95% CI 1.14–1.53; *p* < 0.01; I²=0%; Figure S1), 12 months (RR 2.15; 95% CI 1.56–2.95; *p* < 0.01; I²=10%; Figure S1), 18 months (RR 3.54; 95% CI 2.24–5.59; *p* < 0.01; I²=0%; Figure S1), 24 months (RR 3.58; 95% CI 1.81–7.10; *p* < 0.01; I²=0%; Figure S1), and 30 months (RR 3.13; 95% CI 1.26–7.78; *p* = 0.01; I²=0%; Figure S1).

In patients with pMMR tumors, there was no significant difference from baseline in PFS at 6 months (RR 1.09; 95% CI 0.97–1.22; *p* = 0.14; I²=0%; Figure S2), 12 months (RR 1.23; 95% CI 0.95–1.60; *p* = 0.11; I²=12%; Figure S2), 18 months (RR 1.34; 95% CI 0.80–2.26; *p* = 0.27; I²=44%; Figure S2), 24 months (RR 1.30; 95% CI 0.64–2.64; *p* = 0.47; I²=24%; Figure S2), and 30 months (RR 2.24; 95% CI 0.79–6.39; *p* = 0.13; I²=0%; Figure S2).

### Overall survival

**Fig. 3:**
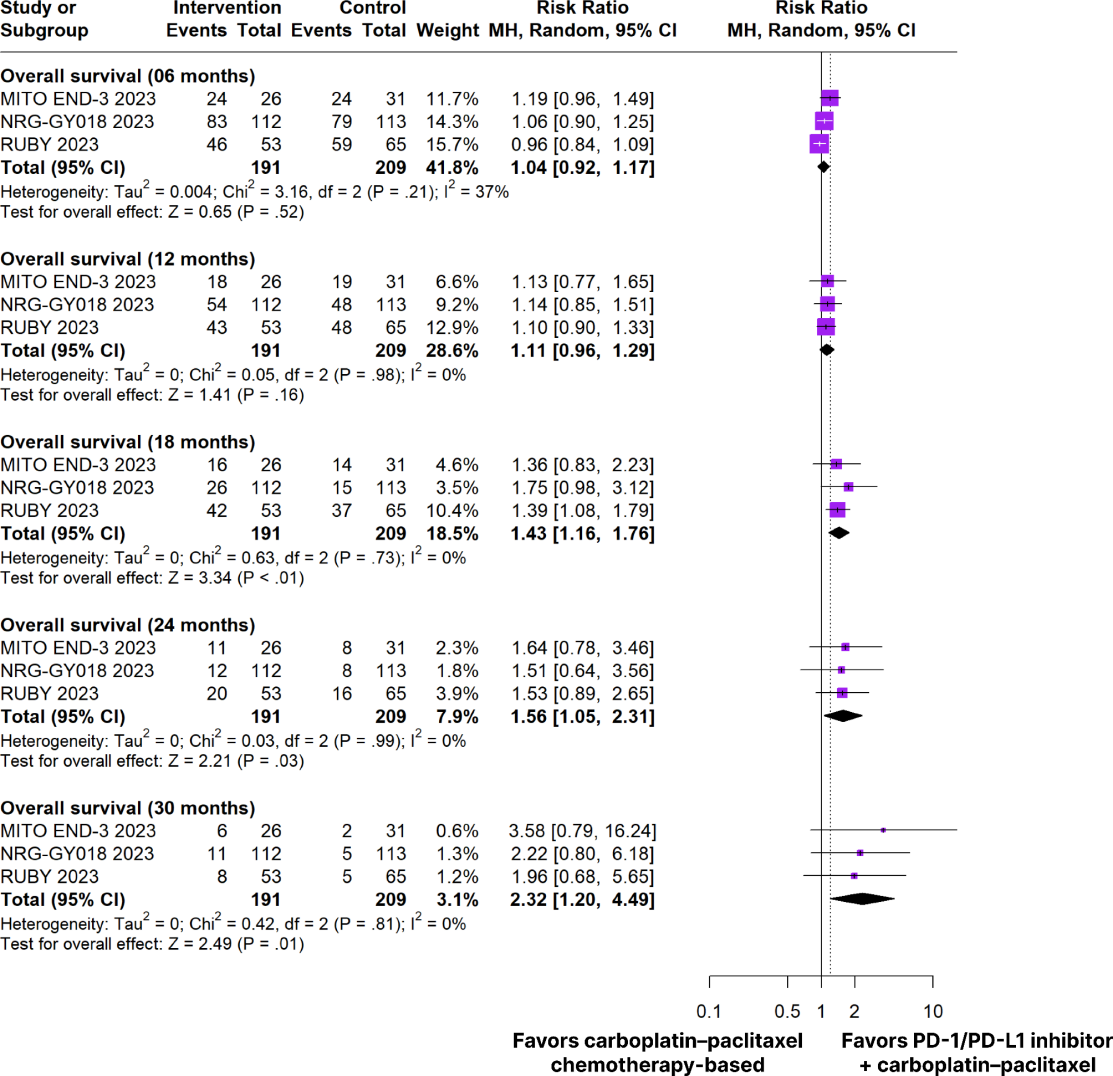
Overall survival of patients with dMMR endometrial cancer treated with PD-1/PD-L1 inhibitors plus carboplatin and paclitaxel chemotherapy-based versus carboplatin plus paclitaxel chemotherapy-based

In patients with dMMR tumors, there was a significant difference from baseline in favor of the PD-1/PD-L1 inhibitors plus carboplatin and paclitaxel chemotherapy-based group in OS at 18 months (RR 1.43; 95% CI 1.16–1.76; *p* < 0.01; I²=0%; Figs. 3), 24 months (RR 1.56; 95% CI 1.05–2.31; *p* = 0.03; I²=0%; Figs. 3) and 30 months (RR 2.32; 95% CI 1.20–4.49; *p* = 0.01; I²=0%; Fig. 3). There were no significant differences in OS at 6 months (RR 1.04; 95% Cl 0.92–1.17; *p* = 0.52; I²=37%; Figs. 3) and 12 months (RR 1.11; 95% Cl 0.96–1.29; *p* = 0.16; I²=0%; Fig. 3).

**Fig. 4:**
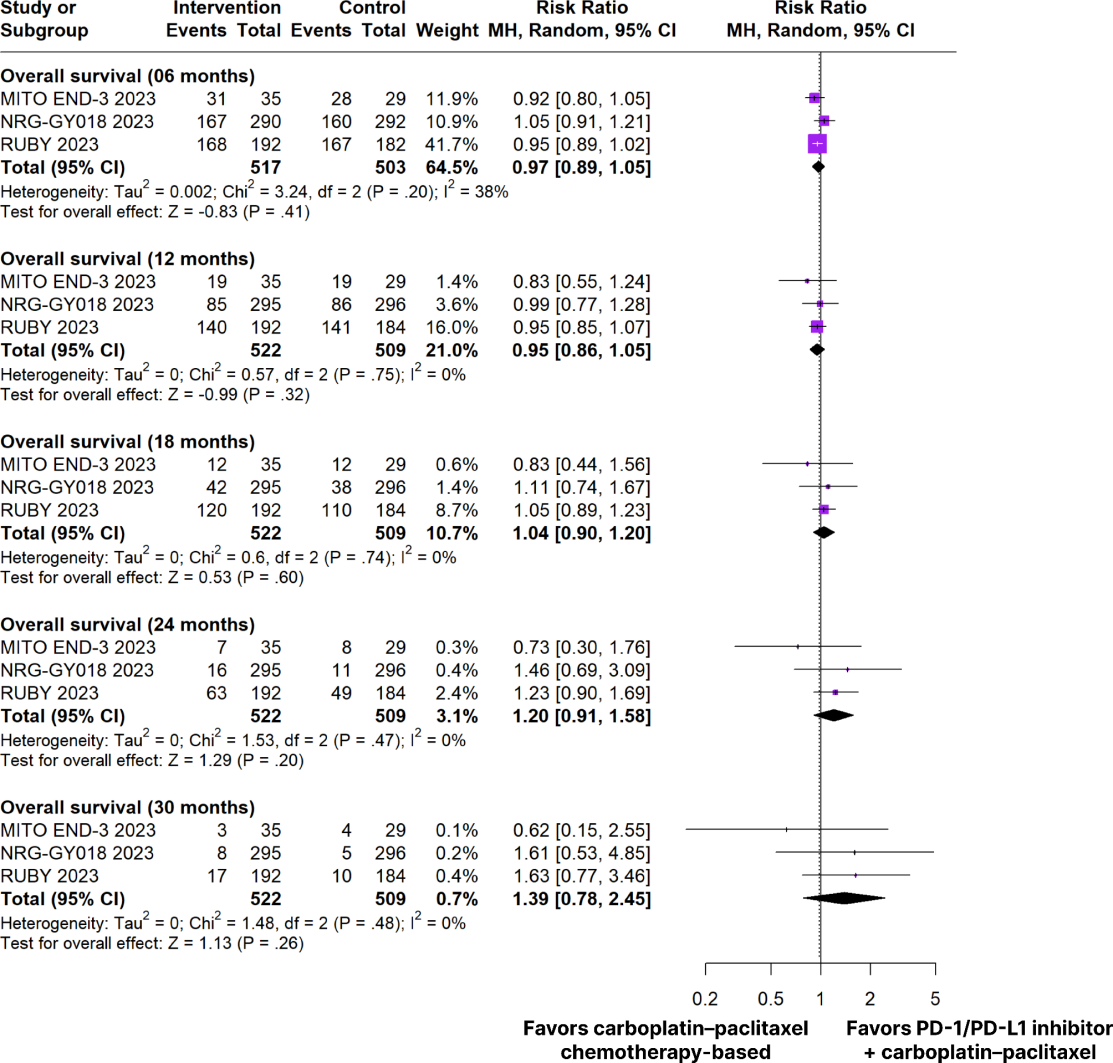
Overall survival of patients with pMMR endometrial cancer treated with PD-1/PD-L1 inhibitors plus carboplatin and paclitaxel chemotherapy-based versus carboplatin plus paclitaxel chemotherapy-based

In patients with pMMR tumors, there was no significant difference from baseline to PD-1/PD-L1 inhibitors plus carboplatin and paclitaxel chemotherapy-based compared to carboplatin plus paclitaxel chemotherapy-based OS at 6 months of treatment (RR 0.97; 95% Cl 0.89–1.05; *p* = 0.41; I²=38%; Figs. 4), 12 months (RR 0.95; 95% Cl 0.86–1.05; *p* = 0.32; I²=0%; Figs. 4), 18 months (RR 1.04; 95% Cl 0.90–1.20; *p* = 0.60; I²=0%; Figs. 4), 24 months (RR 1.20; 95% Cl 0.91–1.58; *p* = 0.20; I² =0%; Fig. 4), and 30 months (RR 1.39; 95% CI 0.78–2.45; *p* = 0.26; I²=0%; Fig. 4).

### Adverse effects

In patients with any grade of adverse events, there was a significant increase in nausea (RR 1.13; 95% Cl 1.01–1.27; *p* = 0.04; I²=0%; Figure S3) and rash (RR 1.64; 95% Cl 1.26–2.13; *p* < 0.01; I²=0%; Figure S3) in patients treated with PD-1/PD-L1 inhibitors plus carboplatin and paclitaxel chemotherapy-based. There were no significant differences between groups in fatigue (RR 1.08; 95% Cl 0.93–1.25; *p* = 0.31; I²=48%; Figure S3), peripheral sensory neuropathy (RR 1.0; 95% Cl 0.90–1.11; *p* = 0.97; I²=0%; Figure S3), constipation (RR 1.09; 95% Cl, 0.95–1.25; *p* = 0.22; I²=0%; Figure S3), diarrhea (RR 1.14; 95% Cl 0.98–1.33; *p* = 0.10; I²=0%; Figure S4), dyspnea (RR 1.09; 95% Cl 0.87–1.36; *p* = 0.45; I²=0%; Figure S4), anemia (RR 1.02; 95% Cl 0.91–1.13; *p* = 0.77; I²=0%; Figure S4), arthralgia (RR 0.95; 95% Cl 0.81–1.13; *p* = 0.57; I²=0%; Figure S4), and neutropenia or neutrophil count decreased (RR 0.76; 95% Cl 0.49–1.20; *p* = 0.24; I²=75%; Figure S4).

In patients with grade ≥ 3 adverse events, there was a significant increase in dyspnea (RR 5.60; 95% Cl 1.45–21.56; *p* = 0.01; I²=0%; Figure S5) in patients treated with PD-1/PD-L1 chemotherapy-based inhibitors plus carboplatin and paclitaxel. There were no significant differences between groups in anemia (RR 1.27; 95% Cl 0.82–1.96; *p* = 0.28; I²=52%; Figure S5), and neutropenia or neutrophil count decreased (RR 0.84; 95% Cl 0.52–1.35; *p* = 0.48; I²=71%; Figure S5).

The incidence of adverse events of any grade or grade ≥ 3 of the included studies are reported in Table 2. The rate of side effects was comparable in both treatment groups within the trials. Overall, fatigue was the most prevalent effect with 800 events (58.06% vs. 53.76%). Regarding the systems analysis, 690 patients had peripheral sensory neuropathy (48.25% vs. 48.19%) as the most frequent nervous disorder, 132 had hypertension (9.96% vs. 8.50%) as a cardiovascular disorder, 258 had dyspnea (18.79% vs. 17.27%) as a respiratory disorder, 624 had nausea (46.28% vs. 40.95%) as a gastrointestinal disorder. There was a total of 19 events leading to death (2.10% vs. 0.56%) including cardiac arrest, sepsis, respiratory failure following severe myositis, myelosuppression, hypovolemic shock, opiate overdose, coronavirus disease, general deterioration of physical health, and lower gastrointestinal hemorrhage.

**Table 2:** Any Grade And Grade ≥ 3 Adverse Events Reported In The Included Studies In This Meta-Analysis

Adverse Events	PD-1/PD-L1 inhibitor plus carboplatin-paclitaxel no. (%)		Carboplatin-paclitaxel chemotherapy-based no. (%)	
	(n = 713)		(n = 718)	
(n = 79)	Any Grade n(%)	≥ 3 Grade n(%)	Any Grade n(%)	≥ 3 Grade n(%)
**General disorders and administration site conditions**				
Fatigue	414 (58.06)	7 (0.98)	386 (53.76)	3 (0.42)
Anemia	340 (47.69)	104 (14.59)	339 (47.21)	82 (11.42)
Infusion related reaction	61 (8.55)	8 (1.12)	51 (7.10)	10 (1.39)
Decreased appetite	52 (7.29)	0	43 (5.99)	0
Weight loss	35 (4.91)	2 (0.28)	27 (3.76)	3 (0.42)
Fever	14 (1.96)	0	9 (1.25)	1 (0.14)
Generalized Pain	3 (0.42)	0	5 (0.70)	1 (0.14)
**Nervous system disorders**				
Peripheral sensory neuropathy	344 (48.25)	9 (1.26)	346 (48.19)	6 (0.84)
Paresthesia	17 (2.38)	2 (0.28)	26 (3.62)	1 (0.14)
Stroke	1 (0.14)	1 (0.14)	1 (0.14)	1 (0.14)
Vasovagal reaction	1 (0.14)	1 (0.14)	0	0
**Cardiovascular system disorders**				
Hypertension	71 (9.96)	27 (3.79)	61 (8.50)	11 (1.53)
Supraventricular tachycardia	0	0	1 (0.14)	1 (0.14)
Cardiac disorders (other)	1 (0.14)	1 (0.14)	0	0
**Vascular disorders**				
Pulmonary embolism	6 (0.84)	6 (0.84)	5 (0.70)	5 (0.70)
Thromboembolic event	6 (0.84)	1 (0.14)	2 (0.28)	0
Hot flashes	1 (0.14)	1 (0.14)	2 (0.28)	0
**Respiratory, thoracic and mediastinal disorders**				
Dyspnea	134 (18.79)	14 (1.96)	124 (17.27)	2 (0.28)
Pneumonitis	5 (0.70)	2 (0.28)	3 (0.42)	1 (0.14)
**Gastrointestinal disorders**				
Nausea	330 (46.28)	7 (0.98)	294 (40.95)	5 (0.70)
Constipation	266 (37.31)	5 (0.70)	246 (34.26)	3 (0.42)
Diarrhea	234 (32.82)	12 (1.68)	207 (28.83)	4 (0.56)
Vomiting	86 (12.06)	5 (0.70)	54 (7.52)	5 (0.70)
Colitis	11 (1.54)	0	4 (0.56)	1 (0.14)
Pyrexia	6 (0.84)	6 (0.84)	2 (0.28)	2 (0.28)
Abdominal pain	7 (0.98)	2 (0.28)	3 (0.42)	0
Pancreatitis	1 (0.14)	0	4 (0.56)	0
Mucositis oral	2 (0.28)	0	3 (0.42)	0
Ascites	1 (0.14)	1 (0.14)	0	0
Hepatic Failure	1 (0.14)	1 (0.14)	0	0
Colonic obstruction	0	0	1 (0.14)	1 (0.14)
**Renal and urinary disorders**				
Urinary tract infection	4 (0.56)	4 (0.56)	7 (0.97)	5 (0.70)
Acute Kidney Injury	7 (0.98)	7 (0.98)	3 (0.42)	3 (0.42)
Urinary tract obstruction	1 (0.14)	1 (0.14)	0	0
Bladder infecction	1 (0.14)	0	1 (0.14)	0
**Skin and subcutaneous tissue disorders**				
Alopecia	145 (20.34)	0	149 (20.75)	0
Rash	125 (17.53)	7 (0.98)	77 (10.72)	4 (0.56)
Pruritus	5 (0.70)	1 (0.14)	2 (0.28)	0
Erythema multiforme	2 (0.28)	1 (0.14)	1 (0.14)	0
**Musculoskeletal and connective tissue disorders**				
Arthralgia	191 (26.79)	3 (0.42)	203 (28.27)	3 (0.42)
Myalgia	137 (19.21)	2 (0.28)	133 (18.52)	5 (0.70)
Asthenia	2 (0.28)	2 (0.28)	6 (0.84)	6 (0.84)
Muscular weakness	5 (0.70)	5 (0.70)	1 (0.14)	1 (0.14)
Myositis	2 (0.28)	0	1 (0.14)	1 (0.14)
Bone pain	1 (0.14)	0	3 (0.42)	0
Musculoskeletal and connective tissue disorders (other)	1 (0.14)	1 (0.14)	0	0
**Endocrine system disorders**				
Hypothyroidism	112 (15.71)	27 (3.78)	79 (11.00)	7 (0.97)
Hyperthyroidism	26 (3.65)	0	11 (1.53)	0
Adrenal Insuffiency	4 (0.56)	0	1 (0.14)	0
Hypophysitis	2 (0.28)	2 (0.28)	0	0
**Metabolism and nutrition disorders**				
Hypomagnesemia	52 (7.29)	0	70 (9.75)	0
Hypokalemia	12 (1.68)	0	9 (1.25)	0
Hyperglycaemia	5 (0.70)	2 (0.28)	2 (0.28)	0
Anorexia	2 (0.28)	1 (0.14)	5 (0.70)	0
Hypertriglyceridemia	4 (0.56)	1 (0.14)	0	0
Hypercalcemia	2 (0.28)	0	2 (0.28)	0
Glucose Intolerance	2 (0.28)	0	0	0
**Reproductive system and breast disorders**				
Uterine hemorrhage	4 (0.56)	0	1 (0.14)	1 (0.14)
Pelvic pain	1 (0.14)	1 (0.14)	2 (0.28)	0
Vaginal hemorrhage	2 (0.28)	1 (0.14)	0	0
Reproductive system and breast disorders (other)	1 (0.14)	1 (0.14)	0	0
**Blood and lymphatic system disorders**				
Neutropenia or Neutrophil count decreased	154 (21.60)	110 (15.43)	172 (23.96)	104 (14.48)
Thrombocytopenia	137 (19.21)	20 (2.80)	106 (14.76)	9 (1.25)
White blood cell decreased	36 (5.05)	23 (3.23)	30 (4.17)	21 (2.92)
Alanine aminotransferase increased	22 (3.08)	1 (0.14)	4 (0.56)	0
Platelet count decreased	16 (2.24)	5 (0.70)	17 (2.37)	3 (0.42)
Alkaline phosphatase increased	6 (0.84)	3 (0.42)	1 (0.14)	0
Aspartate aminotransferase increased	7 (0.98)	1 (0.14)	2 (0.28)	0
Creatinine increased	4 (0.56)	0	4 (0.56)	1 (0.14)
Gamma-glutamyl transferase increased	5 (0.70)	1 (0.14)	2 (0.28)	0
Blood bilirubin increased	2 (0.28)	1 (0.14)	0	0
Leukocytosis	0	0	1 (0.14)	1 (0.14)
**Immune system disorders**				
Allergic reaction	8 (1.12)	4 (0.56)	6 (0.84)	1 (0.14)
Immune system disorders (other)	1 (0.14)	1 (0.14)	0	0
**Infections and infestations**				
Tooth infection	1 (0.14)	1 (0.14)	0	0
Wound complication	1 (0.14)	1 (0.14)	0	0
Infections and infestations (other)	5 (0.70)	2 (0.28)	2 (0.28)	0

### Sensitivity analyses

We performed a leave-one-out sensitivity analysis for all outcomes. There was no significant difference in the OS at 24 months in patients with dMMR tumors omitting MITO END-3 or RUBY trials [30, 31]. There was no significant difference in the OS at 30 months in patients with dMMR tumors omitting MITO END-3 or NRG-GY018 trials [29, 31]. There was no significant difference in the PFS at 30 months in patients with dMMR tumors omitting NRG-GY018 trial [29]. There was a significant increase in PFS at 12 and 18 months in patients with pMMR tumors treated with PD-1/PD-L1 inhibitors plus carboplatin and paclitaxel chemotherapy-based omitting MITO END-3 trial [31]. There was a significant increase in PFS analyzed with HR in patients with pMMR tumors treated with PD-1/PD-L1 inhibitors plus carboplatin and paclitaxel chemotherapy-based omitting MITO END-3 trial [31].

In patients with any grade of adverse events, there was a significant increase in fatigue in the intervention group omitting RUBY trial; there was no significant difference in nausea omitting NRG-GY018 or RUBY trials; there was a significant reduction in neutropenia or neutrophil count decreased in the intervention group omitting NRG-GY018 trial [29, 30].

In patients with grade ≥ 3 adverse events, there was a significant increase in anemia in the intervention group omitting RUBY trial; there was no significant difference in dyspnea omitting NRG-GY018 trial; there was a significant reduction in neutropenia or neutrophil count decreased in the intervention group omitting NRG-GY018 trial [29, 30]. Leave-one-out sensitivity analysis of the main outcomes is detailed in Figure S6.

### Quality assessment

**Fig. 5:**
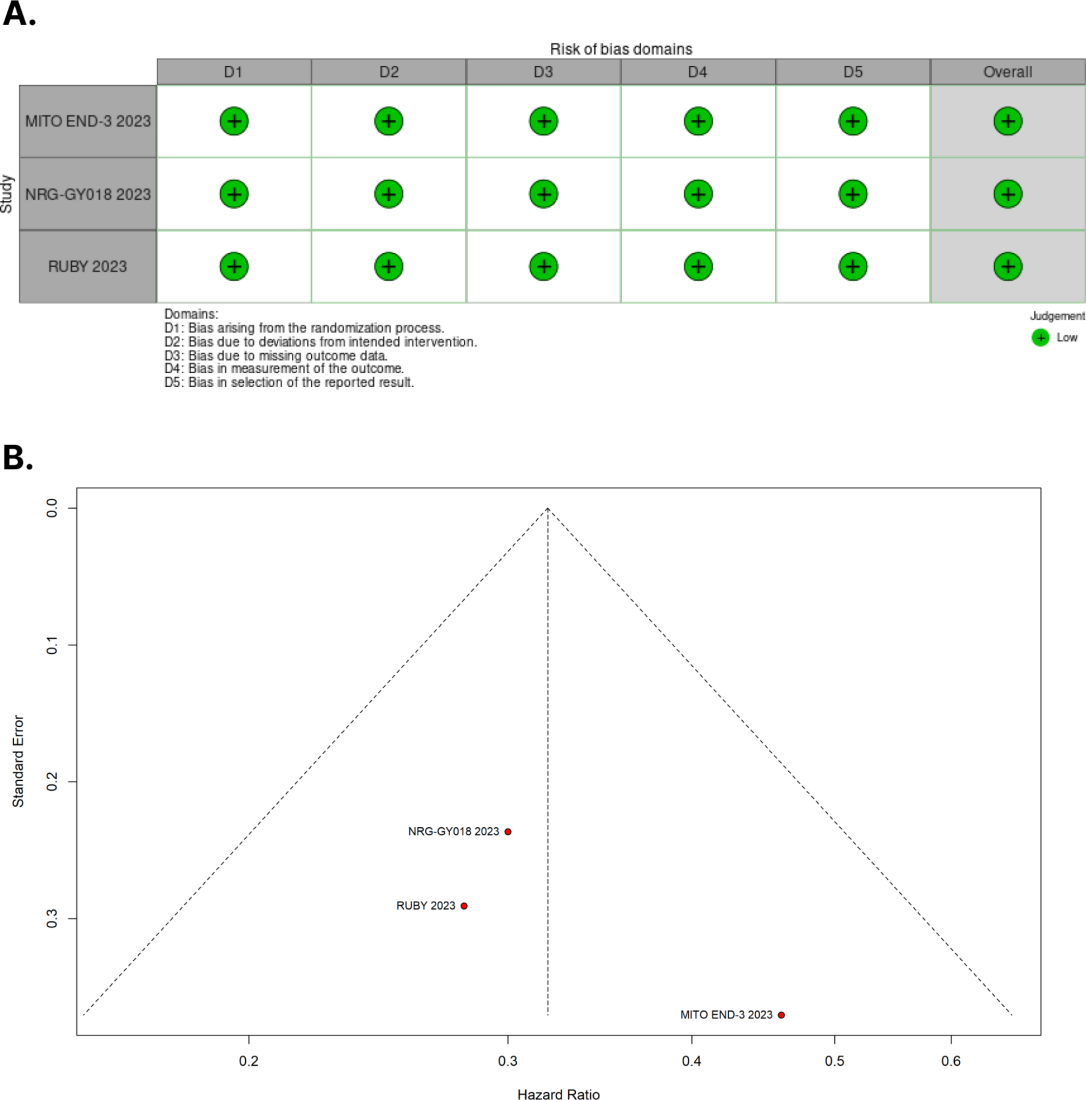
**A.** Critical appraisal of RCTs according to the Cochrane Collaboration’s tool for assessing risk of bias in randomized trials. **B.** Funnel plot analysis of the progression-free survival of patients with dMMR (mismatch repair–deficient) endometrial cancer shows no evidence of publication bias

The individual assessment of each RCT included in the meta-analysis is depicted in Fig. 5A. Overall, all RCTs were deemed at low risk of bias. The symmetrical distribution of comparable studies depicted in the funnel plot in Fig. 5B suggests the absence of publication bias.

## Discussion

In this systematic review and meta-analysis involving 3 RCTs and 1,431 patients, we compared carboplatin and paclitaxel plus PD-1/PD-L1 inhibitors against carboplatin and paclitaxel olone for patients with primary advanced or recurrent endometrial cancer. The main findings from the pooled analyses were as follows: (1) PFS was better in patients receiving PD-1/PD-L1, especially within the subgroup of patients with dMMR; (2) OS showed a significant difference favoring PD-1/PD-L1 group beyond 18 months in dMMR subgroup, while no difference was observed for patients with pMMR; and (3) adverse effects such as nausea, rash, fatigue, peripheral neuropathy, constipation, diarrhea, dyspnea, anemia, neutropenia, arthralgia, and hypothyroidism were noted in both treatment groups.

Inhibition of T cells via PD-1/PD-L1 has demonstrated marked benefits in solid tumors, including melanoma and non-small cell lung cancer. The quantification of this biomarker may help select patients who may derive the most benefit from immunotherapies [32–34]. In the immunohistochemical analysis of 1,599 samples from gynecological cancers, PD-1 expression occurred in 67.9% (1,086) and PD-L1 in 19.6% (313). Notably, among these tumors, the highest rate of direct expression of PD-1 occurred in endometrial cancer (343/456), with 75.2%, whereas PD-L1 was found in only 25.2% of cases (115/456) [35].

Metastatic endometrial cancers with DNA dMMR/MSI-H tend to respond better to inhibitors of PD-1 and its ligand, PD-L1, than those with pMMR tumors [36]. These drugs are a negative regulator of T-cell activation and proliferation and prevent excessive immune reaction and autoimmunity, known as ICIs. Anti–PD–1 receptor monoclonal antibody checkpoint inhibitor blocks the inhibitory pathway and allows increased immunogenicity of tumors. This aligns with our study regarding PFS pointing in favor of the PD-1 plus carboplatin-paclitaxel with over 68% higher PFS, especially in the dMMR subgroup analysis when compared with carboplatin-paclitaxel only [37]. Furthermore, it also takes in favor of the finding that the PFS in the pMMR tumors subgroup analysis had no significant difference [38].

The response to therapy with ICIs depends on MSI and dMMR. It is present in Lynch syndrome and sporadic cancers, being colorectal, gastric, small intestine, urothelial, central nervous system, and sebaceous gland neoplasms [12]. About 15% of colorectal cancers have genetic instability, resulting in dMMR status [39]. This high tumor mutational burden is directly related to the production of tumor neoantigens, consequently generating a pro-inflammatory tumor microenvironment, and this is why dMMR tumors respond well to immunotherapies [40, 41]. The Canadian Cancer Trials Group CO.26 study evaluated durvalumab versus the best supportive care for patients with refractory metastatic colorectal cancer, although no prolongation for median PFS (1.8 months vs. 1.9 months) the median OS goes better in the immunotherapy group (6.6 months vs. 4.1 months) [42]. In contrast, our meta-analysis supports that the addition of immunotherapy in endometrial cancer prolonged PFS overall and for the dMMR population.

Patients with endometrial tumors expressing PD-L1 show a tendency towards improved OS [43]. However, this result has previously been described in Merkel cell carcinoma, melanoma, and pMMR colorectal carcinoma [32, 44, 45]. PD-L1 upregulation is driven by activation of the IFNγ pathway and CD8 + T cells. Thus, this activation may represent an ongoing antitumor response, a negative feedback dependent on an infiltrating immune response [46].

In our meta-analysis, the OS had a significant difference when over 18 months of treatment. To our knowledge, this is due to the short time of treatment cut-off analysis between 0 and 12 months. Looking up to the dMMR/MSI-H group there was a better OS at 18, 24, and 30 months compared to the pMMR at the same period with the dMMR group having a significant difference against no significant findings in the pMMR group at any period of follow-up pointing to the consolidate literature about lack of therapy response in the pMMR group due to more immune stability [37, 47]. Moreover, these findings are in line with the literature that dMMR group status by immunohistochemistry is associated with a higher response rate to immunotherapy, leading to better OS rates, especially with 30-month follow-up [36, 38].

Adverse events on overall well-being associated with the chosen pharmacotherapy generally have a detrimental influence on the patient’s daily life, compromising their routine activities and emotional state. Although the frequency of adverse events is commonly higher in combined chemotherapies, only nausea and cutaneous rash showed a statistically significant difference between groups, both were increased in the group treated with PD-1/PD-L1 inhibitors plus chemotherapy [36, 38]. Nonetheless, considering the overall benefit achieved with the addition of Anti-PD1, the significant adverse events reached grade 1 on the severity scale, meaning they were not worsened compared to the placebo.

This study has some limitations. First, the analysis was based on a restricted (limited) number of RCTs, which may influence the effect size found in our results. However, the absence of heterogeneity in the pooled analysis of the majority of outcomes suggests that our meta-analysis conveys the best available evidence for the use of PD-1/PD-L1 inhibitors plus carboplatin and paclitaxel as a treatment for primary advanced or recurrent endometrial cancer. The heterogeneity of the PFS outcome may be associated with different populations with pMMR and dMMR tumors evaluated in subgroups and together, different trial phases, and differences between the drugs used. Second, the absence of data did not allow for the reporting of other outcomes of interest, such as overall response rate, complete response, partial response, and stable disease. Third, studies from different phases were included. However, they are essential to elucidate the most current evidence on the addition of PD-1/PD-L1 inhibitors to chemotherapy in the treatment of endometrial cancer. Fourth, the included studies presented different follow-ups, which may influence the effect sizes of our results. Finally, one RCT did not report HR for OS, making it impossible to analyze this outcome with HR. However, this did not prevent robust conclusions about the outcomes analyzed in each group.

Therefore, considering the limitations of our meta-analysis and the current role of immunotherapeutics in endometrial cancer, future research is needed to explore the role of immunotherapy alone for patients with dMMR tumors. Considering the approval of immunotherapeutics as second-line after progression to chemotherapy, investigating the efficacy of immunotherapy as a first-line option has considerable potential. Successful results in this context could spare patients from chemotherapy toxicity, making this a critical area for further exploration. Furthermore, this approach could offer a valuable alternative for patients who are not eligible for cytotoxic chemotherapy, including comorbidities or treatment intolerance.

## Conclusion

This is the first meta-analysis of RCTs to evaluate first-line immunotherapy for advanced or recurrent endometrial cancer. Our results support that the addition of PD-1/PD-L1 inhibitors to chemotherapy is associated with significant improvement in PFS, particularly in the dMMR/MSI-H population. The combination is not associated with increased toxicities to the treatment.

### Electronic supplementary material

Below is the link to the electronic supplementary material.

**Supplementary Material: Table S1**. Inclusion and exclusion criteria of included studies. **Table S2**. Search Strategies. **Figure S1**. Progression-free survival of patients with dMMR (mismatch repair?deficient) endometrial cancer treated with PD-1/PD-L1 inhibitors plus carboplatin and paclitaxel chemotherapy-based versus carboplatin plus paclitaxel chemotherapy-based. **Figure S2**. Progression-free survival of patients with pMMR (mismatch repair?proficient) endometrial cancer treated with PD-1/PD-L1 inhibitors plus carboplatin and paclitaxel chemotherapy-based versus carboplatin plus paclitaxel chemotherapy-based. **Figure S3**. Any grade of adverse events. A. Nausea. B. Rash. C. Fatigue. D. Peripheral sensory neuropathy. E. Constipation. **Figure S4**. Any grade of adverse events. A. Diarrhea. B. Dyspnea. C. Anemia. D. Arthralgia. E. Neutropenia or neutrophil count decreased. **Figure S5**. Grade ≥3 adverse events A. Dyspnea. B. Anemia. C. Neutropenia or neutrophil count decreased. **Figure S6**. Leave-one-out sensitivity analyses. A. Progression-free survival of patients with dMMR (mismatch repair?deficient) tumors. B. Progression-free survival of patients with pMMR (mismatch repair?proficient) tumors. C. Overall survival at 30 months of patients with dMMR (mismatch repair?deficient) tumors. D. Overall survival at 30 months of patients with pMMR (mismatch repair?proficient) tumors.

## Data Availability

All data generated and/or analysed during this study are included in this published article [and its supplementary information files].

## Abbreviations

AEs: Adverse events
Cls: Confidence intervals
DC: Dendritic cell
dMMR: Mismatch repair deficiency
FDA: Food and Drug Administration
HRs: Hazard ratio
ICIs: Immune Checkpoint Inhibitors
MSI-H: Microsatellite instability-high
OS: Overall survival
RCTs: Randomized controlled trials
RR: Risk ratio
PD-1: Programmed cell death protein 1
PD-L1: Programmed death ligand 1
PD-L2: Programmed death ligand 2
PFS: Progression-free survival
pMMR: Mismatch repair–proficient
TCD8 +: Cytotoxic T lymphocytes
T-reg: Regulatory T cells
