# Supplementary material for: PD-1/PD-L1 inhibitors plus carboplatin and paclitaxel compared with carboplatin and paclitaxel in primary advanced or recurrent endometrial cancer: a systematic review and meta-analysis of randomized clinical trials
Source: BMC Cancer. 2023 Nov 29;23:1166. doi: 10.1186/s12885-023-11654-z (PMC10688003; doi:10.1186/s12885-023-11654-z)
Supplement: Supplementary file 1 — Supplementary Material: Table S1. Inclusion and exclusion criteria of included studies. Table S2. Search Strategies. Figure S1. Progression-free survival of patients with dMMR (mismatch repair?deficient) endometrial cancer treated with PD-1/PD-L1 inhibitors plus carboplatin and paclitaxel chemotherapy-based versus carboplatin plus paclitaxel chemotherapy-based. Figure S2. Progression-free survival of patients with pMMR (mismatch repair?proficient) endometrial cancer treated with PD-1/PD-L1 inhibitors plus carboplatin and paclitaxel chemotherapy-based versus carboplatin plus paclitaxel chemotherapy-based. Figure S3. Any grade of adverse events. A. Nausea. B. Rash. C. Fatigue. D. Peripheral sensory neuropathy. E. Constipation. Figure S4. Any grade of adverse events. A. Diarrhea. B. Dyspnea. C. Anemia. D. Arthralgia. E. Neutropenia or neutrophil count decreased. Figure S5. Grade ≥3 adverse events A. Dyspnea. B. Anemia. C. Neutropenia or neutrophil count decreased. Figure S6. Leave-one-out sensitivity analyses. A. Progression-free survival of patients with dMMR (mismatch repair?deficient) tumors. B. Progression-free survival of patients with pMMR (mismatch repair?proficient) tumors. C. Overall survival at 30 months of patients with dMMR (mismatch repair?deficient) tumors. D. Overall survival at 30 months of patients with pMMR (mismatch repair?proficient) tumors. [file 12885_2023_11654_MOESM1_ESM.docx]

**Supplementary Material**

**Table S1.** Inclusion and exclusion criteria of included studies.

**Table S2.** Search Strategies.

**Figure S1.** Progression-free survival of patients with dMMR (mismatch repair–deficient) endometrial cancer treated with PD-1/PD-L1 inhibitors plus carboplatin and paclitaxel chemotherapy-based versus carboplatin plus paclitaxel chemotherapy-based.

**Figure S2.** Progression-free survival of patients with pMMR (mismatch repair–proficient) endometrial cancer treated with PD-1/PD-L1 inhibitors plus carboplatin and paclitaxel chemotherapy-based versus carboplatin plus paclitaxel chemotherapy-based.

**Figure S3.** Any grade of adverse events. **A.** Nausea. **B.** Rash. **C.** Fatigue. **D.** Peripheral sensory neuropathy. **E.** Constipation.

**Figure S4.** Any grade of adverse events. **A.** Diarrhea. **B.** Dyspnea. **C.** Anemia. **D.** Arthralgia. **E.** Neutropenia or neutrophil count decreased.

**Figure S5.** Grade ≥3 adverse events **A.** Dyspnea. **B.** Anemia. **C.** Neutropenia or neutrophil count decreased.

**Figure S6.** Leave-one-out sensitivity analyses. **A.** Progression-free survival of patients with dMMR (mismatch repair–deficient) tumors. **B.** Progression-free survival of patients with pMMR (mismatch repair–proficient) tumors. **C.** Overall survival at 30 months of patients with dMMR (mismatch repair–deficient) tumors. **D.** Overall survival at 30 months of patients with pMMR (mismatch repair–proficient) tumors.

**Table S1.** Inclusion and exclusion criteria of included studies

| Study | Inclusion Criteria | Exclusion Criteria |
| --- | --- | --- |
| NRG-GY018 trial (NCT03914612) | 1. Measurable stage III, measurable stage IVA, stage IVB (with or without measurable disease) or recurrent (with or without measurable disease) endometrial cancer. 2. Pathology report showing results of institutional MMR IHC testing. 3. Histologic confirmation of the original primary tumor is required (submission of pathology report(s) is required). Patients with the following histologic types are eligible: Endometrioid adenocarcinoma, serous adenocarcinoma, dedifferentiated/undifferentiated carcinoma, clear cell adenocarcinoma, mixed epithelial carcinoma, adenocarcinoma not otherwise specified (N.O.S.). 4. Submission of tumor specimens for centralized MMR IHC testing is required after Step 1 and before Step 2 registration. 5. In patients with measurable disease, lesions will be defined and monitored by RECIST version (v) 1.1. Measurable disease is defined as at least one lesion that can be accurately measured in at least one dimension (longest diameter to be recorded). Each lesion must be >= 10 mm when measured by computed tomography (CT) or magnetic resonance imaging (MRI). Lymph nodes must be >= 15 mm in short axis when measured by CT or MRI. 6. Patients may have received    1. NO prior chemotherapy for treatment of endometrial cancer OR    2. Prior adjuvant chemotherapy (e.g., paclitaxel/carboplatin alone or as a component of concurrent chemotherapy and radiation therapy [with or without cisplatin]) provided adjuvant chemotherapy was completed >= 12 months prior to STEP 2 registration. 7. Patients may have received prior radiation therapy for treatment of endometrial cancer. Prior radiation therapy may have included pelvic radiation therapy, extended field pelvic/para aortic radiation therapy, intravaginal brachytherapy and/or palliative radiation therapy. All radiation therapy must be completed at least 4 weeks prior to STEP 2 registration. 8. Patients may have received prior hormonal therapy for treatment of endometrial cancer. All hormonal therapy must be discontinued at least three weeks prior to STEP 2 registration. 9. Interval or cytoreductive surgery, after start of treatment on this trial, and prior to documentation of disease progression, is NOT permitted. 10. Age >= 18 11. Performance status of 0, 1 or 2. 12. Platelets >= 100,000/mcl. 13. Absolute neutrophil count (ANC) >= 1,500/mcl. 14. Creatinine =< 1.5 x institutional/laboratory upper limit of normal (ULN). 15. Total serum bilirubin level =< 1.5 x upper limit of normal (ULN) (patients with known Gilbert's disease who have bilirubin level =< 3 x ULN may be enrolled). 16. Aspartate aminotransferase (AST) and alanine aminotransferase (ALT) =< 3 x ULN. 17. Thyroid stimulating hormone (TSH) within normal limits. If TSH is not within normal range despite no symptoms of thyroid dysfunction, normal Free T4 level is required. 18. Human immunodeficiency virus (HIV)-infected patients on effective anti-retroviral therapy with undetectable viral load within 6 months of Step 2 registration are eligible for this trial. 19. For patients of child bearing potential: negative urine or serum pregnancy test. If the urine test is positive or cannot be confirmed as negative, a serum pregnancy test is required. 20. Administration of study drugs (pembrolizumab [MK-3475], paclitaxel, carboplatin) may have an adverse effect on pregnancy and poses a risk to the human fetus, including embryo-lethality. Women of childbearing potential (WOCBP) must agree to use adequate contraception (hormonal or barrier method of birth control; abstinence) from at least 14 days prior to Step 2 registration (for oral contraceptives), during treatment, and for 120 days after the last dose of study medication. Should a woman become pregnant or suspect she is pregnant while she is participating in this study, she should inform her treating physician immediately. Patients will be considered of nonreproductive potential if they are either:     1. Postmenopausal (defined as at least 12 months with no menses without an alternative medical cause; in women < 45 years of age, a high follicle stimulating hormone (FSH) level in the postmenopausal range may be used to confirm a postmenopausal state in women not using hormonal contraception or hormonal replacement therapy. In the absence of 12 months of amenorrhea, a single FSH measurement is insufficient); OR     2. Have a hysterectomy and/or bilateral oophorectomy, bilateral salpingectomy or bilateral tubal ligation/occlusion, at least 6 weeks prior to Step 2 registration; OR     3. Have a congenital or acquired condition that prevents childbearing. 21. Patients with a prior or concurrent malignancy whose natural history or treatment does not have the potential to interfere with the safety or efficacy assessment of the investigational regimen are eligible for this trial. 22. The patient or a legally authorized representative must provide study-specific informed consent prior to study entry and, for patients treated in the United States (U.S.), authorization permitting release of personal health information. | 1. Patients with prior treatment with anti-PD-1, anti-PD-L1 or anti-CTLA-4 therapeutic antibody or other similar agents. 2. Patients who have a history of a severe hypersensitivity reaction to monoclonal antibody or pembrolizumab (MK-3475) and/or its excipients; and/or a severe hypersensitivity reaction to paclitaxel and/or carboplatin 3. Patients who are currently participating and receiving cancer-directed study therapy or have participated in a study of an investigational agent and received cancer-directed study therapy within 4 weeks prior to Step 2 registration. 4. Patients who have a diagnosis of immunodeficiency or are receiving systemic steroid therapy or any other form of immunosuppressive therapy within 7 days prior to Step 2 registration.    1. Patients who have received steroids as CT scan contrast premedication may be enrolled.    2. The use of inhaled or topical corticosteroids is allowed.    3. The use of mineralocorticoids (e.g., fludrocortisone) for patients with orthostatic hypotension or adrenocortical insufficiency is allowed.    4. The use of physiologic doses of corticosteroids may be approved after consultation with the study chair. 5. Patients with treated brain metastases are eligible if follow-up brain imaging after central nervous system (CNS)-directed therapy shows no evidence of progression, and they have been off steroids for at least 4 weeks prior to Step 2 registration and remain clinically stable. 6. Patients with active autoimmune disease or history of autoimmune disease that might recur, which may affect vital organ function or require immune suppressive treatment including systemic corticosteroids. This includes, but is not limited to, patients with a history of immune related neurologic disease, multiple sclerosis, autoimmune (demyelinating) neuropathy, Guillain-Barre syndrome, myasthenia gravis; systemic autoimmune disease such as systemic lupus erythematosus (SLE), connective tissue diseases, scleroderma, inflammatory bowel disease (IBD), Crohn's, ulcerative colitis, hepatitis; and patients with a history of toxic epidermal necrolysis (TEN), Stevens-Johnson syndrome, or phospholipid syndrome because of the risk of recurrence or exacerbation of disease. 7. Patients with vitiligo, endocrine deficiencies including type I diabetes mellitus, thyroiditis managed with replacement hormones including physiologic corticosteroids are eligible. 8. Patients with rheumatoid arthritis and other arthropathies, Sjogren's syndrome and psoriasis controlled with topical medication and patients with positive serology, such as antinuclear antibodies (ANA), anti-thyroid antibodies should be evaluated for the presence of target organ involvement and potential need for systemic treatment but should otherwise be eligible. 9. Patients who have a history of (non-infectious) pneumonitis that required steroids, or current pneumonitis. 10. Uncontrolled intercurrent illness including, but not limited to: ongoing or active infection (except for uncomplicated urinary tract infection), interstitial lung disease or active, non-infectious pneumonitis, symptomatic congestive heart failure, unstable angina pectoris, cardiac arrhythmia, or psychiatric illness/social situations that would limit compliance with study requirements. 11. Known clinically significant liver disease, including active viral, alcoholic, or other hepatitis; and cirrhosis.     1. For patients with evidence of chronic hepatitis B virus (HBV) infection, the HBV viral load must be undetectable on suppressive therapy, if indicated.     2. Patients with a history of hepatitis C virus (HCV) infection must have been treated and cured. For patients with HCV infection who are currently on treatment, they are eligible if they have an undetectable HCV viral load. 12. Pregnant or lactating patients. |
| RUBY trial (NCT03981796) | Part 1 and Part 2:   1. Female participant is at least 18 years of age. 2. Participant has histologically or cytologically proven endometrial cancer with recurrent or advanced disease. 3. Participant must have primary Stage III or Stage IV disease or first recurrent endometrial cancer with a low potential for cure by radiation therapy or surgery alone or in combination and meet at least one of the following criteria;    1. Participant has primary Stage IIIA to IIIC1 disease with presence of evaluable or measurable disease per Response Evaluation Criteria in Solid Tumors (RECIST) version (v).1.1 based on Investigator's assessment. Lesions that are equivocal or can be representative of post-operative change should be biopsied and confirmed for the presence of tumor;    2. Participant has primary Stage IIIC1 disease with carcinosarcoma, clear cell, serous, or mixed histology (containing greater than or equal to [>=] 10 percent carcinosarcoma, clear cell, or serous histology) regardless of presence of evaluable or measurable disease on imaging;    3. Participant has primary Stage IIIC2 or Stage IV disease regardless of the presence of evaluable or measurable disease;    4. Participant has first recurrent disease and is naïve to systemic anticancer therapy;    5. Participant has received prior neo-adjuvant/adjuvant systemic anticancer therapy and had a recurrence or progression of disease (PD) >=6 months after completing treatment (first recurrence only). 4. Participant has an ECOG performance status of 0 or 1. 5. Participant has adequate organ function. 6. Part 2 only: 7. Participants must have normal blood pressure (BP) or adequately treated and controlled hypertension (systolic BP lesser than or equal to [<=] 140 millimeter of mercury [mmHg] and diastolic BP <=90 mmHg). 8. Participants must be able to take medication orally, by mouth (PO). | Part 1 and Part 2:   1. Participant has received neo-adjuvant/adjuvant systemic anticancer therapy for primary Stage III or IV disease and:    1. has not had a recurrence or PD prior to first dose on the study OR    2. has had a recurrence or PD within 6 months of completing systemic anticancer therapy treatment prior to first dose on the study. 2. Participant has had >1 recurrence of endometrial cancer. 3. Participant has received prior therapy with an anti-programmed cell death protein 1 (anti-PD-1), anti-PD-ligand 1 (anti-PD-L1), or anti-PD-ligand 2 (anti-PD-L2) agent. 4. Participant has received prior anticancer therapy (chemotherapy, targeted therapies, hormonal therapy, radiotherapy, or immunotherapy) within 21 days or <5 times the half-life of the most recent therapy prior to Study Day 1, whichever is shorter. 5. Participant has a concomitant malignancy, or participant has a prior non-endometrial invasive malignancy who has been disease-free for <3 years or who received any active treatment in the last 3 years for that malignancy. Non-melanoma skin cancer is allowed. 6. Participant has known uncontrolled central nervous system metastases, carcinomatosis meningitis, or both. 7. Participant has not recovered (that is [i.e.], to Grade <=1 or to Baseline) from cytotoxic therapy induced AEs or has received transfusion of blood products (including platelets or red blood cells) or administration of colony-stimulating factors (including granulocyte colony-stimulating factor [G-CSF], granulocyte macrophage colony-stimulating factor [GM-CSF], or recombinant erythropoietin) within 21 days prior to the first dose of study drug. 8. Participant has not recovered adequately from AEs or complications from any major surgery prior to starting therapy. 9. Participant is currently participating and receiving study treatment or has participated in a study of an investigational agent and received study treatment or used an investigational device within 4 weeks of the first dose of treatment. 10. Participant is considered a poor medical risk due to a serious, uncontrolled medical disorder, nonmalignant systemic disease, or active infection requiring systemic therapy. 11. Participant has received, or is scheduled to receive, a live vaccine within 30 days before first dose of study treatment, during study treatment, and for up to 180 days after receiving the last dose of study treatment. 12. Part 2 only: 13. Participant has received prior therapy with a poly (adenosine diphosphate [ADP]-ribose) polymerase (PARP) inhibitor. 14. Participant has clinically significant cardiovascular disease. 15. Participant has any known history or current diagnosis of myelodysplastic syndrome (MDS) or acute myeloid leukemia (AML 16. ). 17. Participant is at increased bleeding risk due to concurrent conditions. 18. Participant has participated in Part 1 of this study |
| MITO END-3 trial (NCT03503786) | 1. Female aged at least 18 years on day of signing informed consent 2. ECOG Performance Status of 0-1 3. Patients with newly diagnosed or recurrent endometrial cancer FIGO stage III-IV and histologically-confirmed (any histology except sarcoma and carcinosarcoma) 4. Patients may have received adjuvant treatment (platinum-based cytotoxic chemotherapy and/or radiotherapy). Patients having received prior chemotherapy must have completed their treatment at least 6 months prior to registration for protocol therapy. Patients having received prior radiotherapy must have completed their treatment at least 28 days prior to registration for protocol therapy 5. Have measurable disease based on RECIST v1.1 criteria 6. Availability of tumor samples for biomarker analysis 7. Endometrial cancer will include all carcinomas, including endometrioid carcinoma, papillary serous carcinoma, clear cell carcinoma 8. Adequate hematological function defined by absolute neutrophil count (ANC) ≥ 1500 × mm3, platelet count ≥ 100,000 × mm3, and hemoglobin ≥ 9 g/dL (may have been transfused) 9. Adequate hepatic function defined by a total bilirubin level ≤ 1.5 × the upper limit of normal (ULN) range and AST and ALT levels ≤ 2.5 × ULN for all subjects (or ≤ 5 x ULN if liver metastases are present) 10. Adequate renal function defined by an estimated creatinine clearance ≥ 50 mL/min according to the Cockcroft-Gault formula or serum creatinine ≤ 1.5 ULN (for local institutional standard method) 11. Alkaline phosphatase < 1.5 x ULN for the institution (if > 1.5 x ULN, then alkaline phosphatase liver fraction must be < 1.5 ULN) 12. Be willing and able to provide written informed consent/assent for the trial 13. Females of childbearing potential must have a negative serum pregnancy test (serum hCG) at screening. Women of childbearing potential are those who have not been surgically sterilized or have not been free from menses for ≥1 year 14. Highly effective contraception for females if the risk of conception exists. (Note: The effects of the trial drug on the developing human fetus are unknown; thus, women of childbearing potential must agree to use 2 highly effective contraception, defined as methods with a failure rate of less than 1 % per year). Highly effective contraception is required at least 28 days prior, throughout and for at least 60 days after Avelumab treatment | 1. Women who are pregnant or lactating 2. Patients with brain metastases, except those meeting the following criteria:    - Brain metastases that have been treated locally and are clinically stable for at least 2 weeks prior to enrollment    - No ongoing neurological symptoms that are related to the brain localization of the disease (sequelae that are a consequence of the treatment of the brain metastases are acceptable)    - patients must be either off steroids or on a stable or decreasing dose of <10mg daily prednisone (or equivalent) 3. Prior Anticancer treatment for advanced disease and/or prior therapy with an anti-PD-1, anti-PD-L1, or anti-PD-L2 agent. Previous hormonal therapy for advanced disease is allowed, but treatment must be discontinued at least 28 days prior to registration for protocol therapy 4. History of anaphylaxis, or uncontrolled asthma (that is, 3 or more features of partially controlled asthma) 5. Known prior severe hypersensitivity to investigational product or any component in its formulations, including known severe hypersensitivity reactions to monoclonal antibodies (NCI CTCAE v4.03 Grade ≥ 3) 6. Prior organ transplantation, including allogeneic stem cell transplantation 7. Significant acute or chronic infections including, among others:    - Known history of testing positive test for human immunodeficiency virus (HIV) or known acquired immunodeficiency syndrome (AIDS)    - Positive test for hepatitis B surface antigen and / or confirmatory hepatitis C RNA (if anti-hepatitis C antibody tested positive)    - Evidence of interstitial lung disease or active non-infectious pneumonitis.    - Active infection requiring systemic therapy    - Known history of active Tuberculosis Bacillus (TB) 8. Active autoimmune disease that might deteriorate when receiving an immunostimulatory agent:    - Subjects with diabetes type I, vitiligo, psoriasis, hypo- or hyperthyroid disease not requiring immunosuppressive treatment are eligible    - Subjects requiring hormone replacement with corticosteroids are eligible if the steroids are administered only for the purpose of hormonal replacement and at doses ≤ 10 mg or 10 mg equivalent prednisone per day    - Administration of steroids through a route known to result in a minimal systemic exposure (topical, intranasal, intro-ocular, or inhalation) are acceptable 9. Persisting toxicity related to prior therapy of Grade >1 NCI-CTCAE v 4.03; however, alopecia and sensory neuropathy Grade ≤ 2 is acceptable 10. Another primary malignancy within the past five years (except for non-melanoma skin cancer and cervical carcinoma in situ). 11. Concurrent treatment with immunosuppressive or investigational agents EXCEPT for the following: a. intranasal, inhaled, topical steroids, or local steroid injection (e.g., intra-articular injection); b. Systemic corticosteroids at physiologic doses ≤ 10 mg/day of prednisone or equivalent; c. Steroids as premedication for hypersensitivity reactions (e.g., CT scan premedication). 12. Active cardiac disease, defined as:     - Myocardial infarction or unstable angina pectoris within 6 months of the first date of study therapy,     - History of serious ventricular arrhythmia (i.e., ventricular tachycardia or ventricular fibrillation), high-grade atrio-ventricular block, or other cardiac arrhythmias requiring anti-arrhythmic medications (except for atrial fibrillation that is well controlled with antiarrhythmic medication); history of QT interval prolongation.     - New York Heart Association (NYHA) Class III or greater congestive heart failure, or left ventricular ejection fraction of < 40%. 13. Known alcohol or drug abuse 14. Vaccination within 4 weeks of the first dose of avelumab and while on trial is prohibited except for administration of inactivated vaccines 15. Any psychiatric condition that would prohibit the understanding or rendering of informed consent 16. All other significant diseases (for example, inflammatory bowel disease, uncontrolled asthma), which, in the opinion of the Investigator, All other significant diseases (for example, inflammatory bowel disease, uncontrolled asthma) including recent or active suicidal ideation or behavior, which, in the opinion of the Investigator, may increase the risk associated with study participation or study treatment administration or may interfere with the interpretation of study results and, in the judgment of the investigator, would make the patient inappropriate for entry into this study. |

**Table S2.** Search Strategies

| **Database** | **Search Strategy** |
| --- | --- |
| **PubMed** | ("Endometrial Neoplasm" OR "Endometrial Neoplasms" OR "endometrial Carcinoma" OR "Endometrial Carcinomas" OR "Endometrial cancer" OR "Endometrial Cancers" OR "Endometrium cancer" OR "Endometrium Neoplasms" OR “Endometrium Neoplasm" OR "Endometrium Cancers" OR "Cancer of the Endometrium" OR "Carcinoma of Endometrium" OR "Endometrium Carcinoma" OR "Endometrium Carcinomas" OR "Cancer of Endometrium" OR "endometrial carcinosarcoma” OR “Endometrioid Carcinoma” OR “Endometrioid Carcinomas” OR “Endometrioid Adenocarcinoma” OR “Endometrioid Adenocarcinomas” OR “Endometrial Stromal Tumor” OR “Endometrial Stromal Tumors” OR “Endometrial Stromal Low-grade sarcoma” OR “Endolymphatic Stromal Myosis” OR “Endolymphatic Stromal Myoses” OR ”Endometrial Stromal Sarcoma” OR “Endometrial Stromal Sarcomas”) AND ("Immune Checkpoint Inhibitors" OR "Immune Checkpoint Inhibitor" OR "Checkpoint Inhibitors" OR "Checkpoint Inhibitor" OR "Immune Checkpoint Blockers" OR "Checkpoint Blockers" OR "Immune Checkpoint Blockade" OR "Checkpoint Blockade" OR "Immune Checkpoint Inhibition" OR "Checkpoint Inhibition" OR "PD-L1 Inhibitors" OR "PD L1 Inhibitors" OR "PD-L1 Inhibitor" OR "PD L1 Inhibitor" OR "Programmed Death-Ligand 1 Inhibitors" OR "Programmed Death Ligand 1 Inhibitors" OR "PD-1/PD-L1 Blockade" OR "Blockade PD-1/PD-L1" OR "PD 1 PD L1 Blockade" OR "PD-1 Inhibitors" OR "PD 1 Inhibitors" OR "PD-1 Inhibitor" OR "PD-1 Inhibitor" OR "PD 1 Inhibitor" OR "Programmed Cell Death Protein 1 Inhibitor" OR "Programmed Cell Death Protein 1 Inhibitors" OR nivolumab OR Pembrolizumab OR Dostarlimab OR Durvalumab OR atezolizumab OR avelumab OR Keytruda OR Opdivo OR Tecentriq OR bevancio OR Imfinzi OR jamperli) |
| **Scopus** | (TITLE-ABS-KEY("Endometrial Neoplasm") OR TITLE-ABS-KEY("Endometrial Neoplasms") OR TITLE-ABS-KEY(“endometrial Carcinoma") OR TITLE-ABS-KEY("Endometrial Carcinomas") OR TITLE-ABS-KEY(“Endometrial cancer”) OR TITLE-ABS-KEY(“Endometrial Cancers”) OR TITLE-ABS-KEY(“Endometrium cancer") OR TITLE-ABS-KEY("Endometrium Neoplasms") OR TITLE-ABS-KEY("Endometrium Neoplasm") OR TITLE-ABS-KEY("Endometrium Cancers") OR TITLE-ABS-KEY("Cancer of the Endometrium") OR TITLE-ABS-KEY(“Carcinoma of Endometrium”) OR TITLE-ABS-KEY("Endometrium Carcinoma") OR TITLE-ABS-KEY("Endometrium Carcinomas") OR TITLE-ABS-KEY("Cancer of Endometrium") OR TITLE-ABS-KEY("endometrial carcinosarcoma") OR TITLE-ABS-KEY("Endometrioid Carcinoma") OR TITLE-ABS-KEY(“Endometrioid Carcinomas”) OR TITLE-ABS-KEY(“Endometrioid Adenocarcinoma”) OR TITLE-ABS-KEY("Endometrioid Adenocarcinomas”) OR TITLE-ABS-KEY("Endometrial Stromal Tumor") OR TITLE-ABS-KEY("Endometrial Stromal Tumors" ) OR TITLE-ABS-KEY ( "Endometrial Stromal Low-grade sarcoma") OR TITLE-ABS-KEY(“Endolymphatic Stromal Myosis”) OR TITLE-ABS-KEY("Endolymphatic Stromal Myoses") OR TITLE-ABS-KEY("Endometrial Stromal Sarcoma") OR TITLE-ABS-KEY("Endometrial Stromal Sarcomas")) AND (TITLE-ABS-KEY("Immune Checkpoint Inhibitors") OR TITLE-ABS-KEY("Immune Checkpoint Inhibitor") OR TITLE-ABS-KEY("Checkpoint Inhibitors") OR TITLE-ABS-KEY("Checkpoint Inhibitor") OR TITLE-ABS-KEY("Immune Checkpoint Blockers") OR TITLE-ABS-KEY("Checkpoint Blockers") OR TITLE-ABS-KEY("Immune Checkpoint Blockade") OR TITLE-ABS-KEY("Checkpoint Blockade") OR TITLE-ABS-KEY("Immune Checkpoint Inhibition") OR TITLE-ABS-KEY(“Checkpoint Inhibition”) OR TITLE-ABS-KEY("PD-L1 Inhibitors") OR TITLE-ABS-KEY(“PD L1 Inhibitors”) OR TITLE-ABS-KEY("PD-L1 Inhibitor") OR TITLE-ABS-KEY("PD L1 Inhibitor") OR TITLE-ABS-KEY("Programmed Death-Ligand 1 Inhibitors") OR TITLE-ABS-KEY("Programmed Death Ligand 1 Inhibitors") OR TITLE-ABS-KEY("PD-1/PD-L1 Blockade") OR TITLE-ABS-KEY(“Blockade PD-1/PD-L1”) OR TITLE-ABS-KEY(“PD 1 PD L1 Blockade”) OR TITLE-ABS-KEY(“PD-1 Inhibitors”) OR TITLE-ABS-KEY(“PD 1 Inhibitors”) OR TITLE-ABS-KEY(“PD-1 Inhibitor”) OR TITLE-ABS-KEY("PD-1 Inhibitor") OR TITLE-ABS-KEY("PD 1 Inhibitor") OR TITLE-ABS-KEY(“Programmed Cell Death Protein 1 Inhibitor”) OR TITLE-ABS-KEY("Programmed Cell Death Protein 1 Inhibitors”) OR TITLE-ABS-KEY(nivolumab) OR TITLE-ABS-KEY(Pembrolizumab) OR TITLE-ABS-KEY(Dostarlimab) OR TITLE-ABS-KEY(Durvalumab) OR TITLE-ABS-KEY(atezolizumab) OR TITLE-ABS-KEY(avelumab) OR TITLE-ABS-KEY(Keytruda) OR TITLE-ABS-KEY(Opdivo) OR TITLE-ABS-KEY(Tecentriq) OR TITLE-ABS-KEY(bevancio) OR TITLE-ABS-KEY(Imfinzi) OR TITLE-ABS-KEY(jamperli)) |
| **Web of Science** | (TS=("Endometrial Neoplasm") OR TS=("Endometrial Neoplasms") OR TS=("endometrial Carcinoma") OR TS=("Endometrial Carcinomas") OR TS=("Endometrial cancer") OR TS= ("Endometrial Cancers") OR TS=("Endometrium cancer") OR TS=("Endometrium Neoplasms") OR TS=("Endometrium Neoplasm") OR TS=("Endometrium Cancers") OR TS=("Cancer of the Endometrium") OR TS=("Carcinoma of Endometrium") OR TS=("Endometrium Carcinoma") OR TS=("Endometrium Carcinomas") OR TS=("Cancer of Endometrium") OR TS=("endometrial carcinosarcoma") OR TS=("Endometrioid Carcinoma") OR TS=("Endometrioid Carcinomas”) OR TS=("Endometrioid Adenocarcinoma") OR TS=(“Endometrioid Adenocarcinomas”) OR TS=("Endometrial Stromal Tumors") OR TS=("Endometrial Stromal Low-grade sarcoma") OR TS=(“Endometrial Stromal Tumor") OR TS=("Endolymphatic Stromal Myosis") OR TS=("Endolymphatic Stromal Myoses") OR TS=("Endometrial Stromal Sarcoma") OR TS=("Endometrial Stromal Sarcomas")) AND (TS=("Immune Checkpoint Inhibitors") OR TS=("Immune Checkpoint Inhibitor”) OR TS=("Checkpoint Inhibitors") OR TS=("Checkpoint Inhibitor") OR TS=("Immune Checkpoint Blockers") OR TS=("Checkpoint Blockers") OR TS=("Immune Checkpoint Blockade") OR TS=("Checkpoint Blockade") OR TS=("Immune Checkpoint Inhibition”) OR TS=("Checkpoint Inhibition") OR TS=("PD-L1 Inhibitors") OR TS=("PD L1 Inhibitors") OR TS=("PD-L1 Inhibitor") OR TS=("PD-L1 Inhibitor") OR TS=("PD L1 Inhibitor") OR TS=("Programmed Death-Ligand 1 Inhibitors") OR TS=("Programmed Death Ligand 1 Inhibitors") OR TS=("PD-1/PD-L1 Blockade") OR TS= ("Blockade PD-1/PD-L1") OR TS=("PD 1 PD L1 Blockade") OR TS= ("PD-1 Inhibitors") OR TS= ("PD 1 Inhibitors") OR TS= ("PD-1 Inhibitor") OR TS=("PD 1 Inhibitor") OR TS=("Programmed Cell Death Protein 1 Inhibitor") OR TS=("Programmed Cell Death Protein 1 Inhibitors") OR TS=(nivolumab) OR TS=(Pembrolizumab) OR TS=(Dostarlimab) OR TS=(Durvalumab) OR TS=(atezolizumab) OR TS=(avelumab) OR TS=(Keytruda) OR TS=(Opdivo) OR TS=(Tecentriq) OR TS=(bevancio) OR TS=(Imfinzi) OR TS=(jamperli)) |
| **The Cochrane Library** | (("Endometrial Neoplasm"):ti,ab,kw OR ("Endometrial Neoplasms"):ti,ab,kw OR ("endometrial Carcinoma"):ti,ab,kw OR ("Endometrial Carcinomas"):ti,ab,kw OR ("Endometrial cancer"):ti,ab,kw OR ("Endometrial Cancers"):ti,ab,kw OR ("Endometrium cancer"):ti,ab,kw OR ("Endometrium Neoplasms"):ti,ab,kw OR ("Endometrium Neoplasm"):ti,ab,kw OR ("Endometrium Cancers"):ti,ab,kw OR ("Cancer of the Endometrium"):ti,ab,kw OR ("Carcinoma of Endometrium"):ti,ab,kw OR ("Endometrium Carcinoma"):ti,ab,kw OR ("Endometrium Carcinomas"):ti,ab,kw OR ("Cancer of Endometrium"):ti,ab,kw OR ("endometrial carcinosarcoma"):ti,ab,kw OR ("Endometrioid Carcinoma"):ti,ab,kw OR ("Endometrioid Carcinomas”):ti,ab,kw OR ("Endometrioid Adenocarcinoma"):ti,ab,kw OR(“Endometrioid Adenocarcinomas”):ti,ab,kw OR ("Endometrial Stromal Tumors"):ti,ab,kw OR ("Endometrial Stromal Low-grade sarcoma"):ti,ab,kw OR (“Endometrial Stromal Tumor"):ti,ab,kw OR ("Endolymphatic Stromal Myosis"):ti,ab,kw OR ("Endolymphatic Stromal Myoses"):ti,ab,kw OR ("Endometrial Stromal Sarcoma"):ti,ab,kw OR ("Endometrial Stromal Sarcomas"):ti,ab,kw) AND (("Immune Checkpoint Inhibitors"):ti,ab,kw OR ("Immune Checkpoint Inhibitor”):ti,ab,kw OR ( "Checkpoint Inhibitors" ):ti,ab,kw OR ("Checkpoint Inhibitor"):ti,ab,kw OR ("Immune Checkpoint Blockers"):ti,ab,kw OR ("Checkpoint Blockers"):ti,ab,kw OR ("Immune Checkpoint Blockade"):ti,ab,kw OR ("Checkpoint Blockade"):ti,ab,kw OR ("Immune Checkpoint Inhibition”):ti,ab,kw OR ("Checkpoint Inhibition"):ti,ab,kw OR ("PD-L1 Inhibitors"):ti,ab,kw OR ("PD L1 Inhibitors"):ti,ab,kw OR ("PD-L1 Inhibitor"):ti,ab,kw OR ("PD-L1 Inhibitor"):ti,ab,kw OR ("PD L1 Inhibitor"):ti,ab,kw OR ("Programmed Death-Ligand 1 Inhibitors"):ti,ab,kw OR ("Programmed Death Ligand 1 Inhibitors"):ti,ab,kw OR ("PD-1/PD-L1 Blockade"):ti,ab,kw OR ("Blockade PD-1/PD-L1"):ti,ab,kw OR ("PD 1 PD L1 Blockade"):ti,ab,kw OR ("PD-1 Inhibitors"):ti,ab,kw OR ("PD 1 Inhibitors"):ti,ab,kw OR ("PD-1 Inhibitor"):ti,ab,kw OR ("PD 1 Inhibitor"):ti,ab,kw OR ("Programmed Cell Death Protein 1 Inhibitor"):ti,ab,kw OR ("Programmed Cell Death Protein 1 Inhibitors"):ti,ab,kw OR (nivolumab):ti,ab,kw OR (Pembrolizumab):ti,ab,kw OR (Dostarlimab):ti,ab,kw OR (Durvalumab):ti,ab,kw OR (atezolizumab):ti,ab,kw OR (avelumab):ti,ab,kw OR (Keytruda):ti,ab,kw OR (Opdivo):ti,ab,kw OR (Tecentriq):ti,ab,kw OR (bevancio):ti,ab,kw OR (Imfinzi):ti,ab,kw OR (jamperli):ti,ab,kw) |
|  |  |


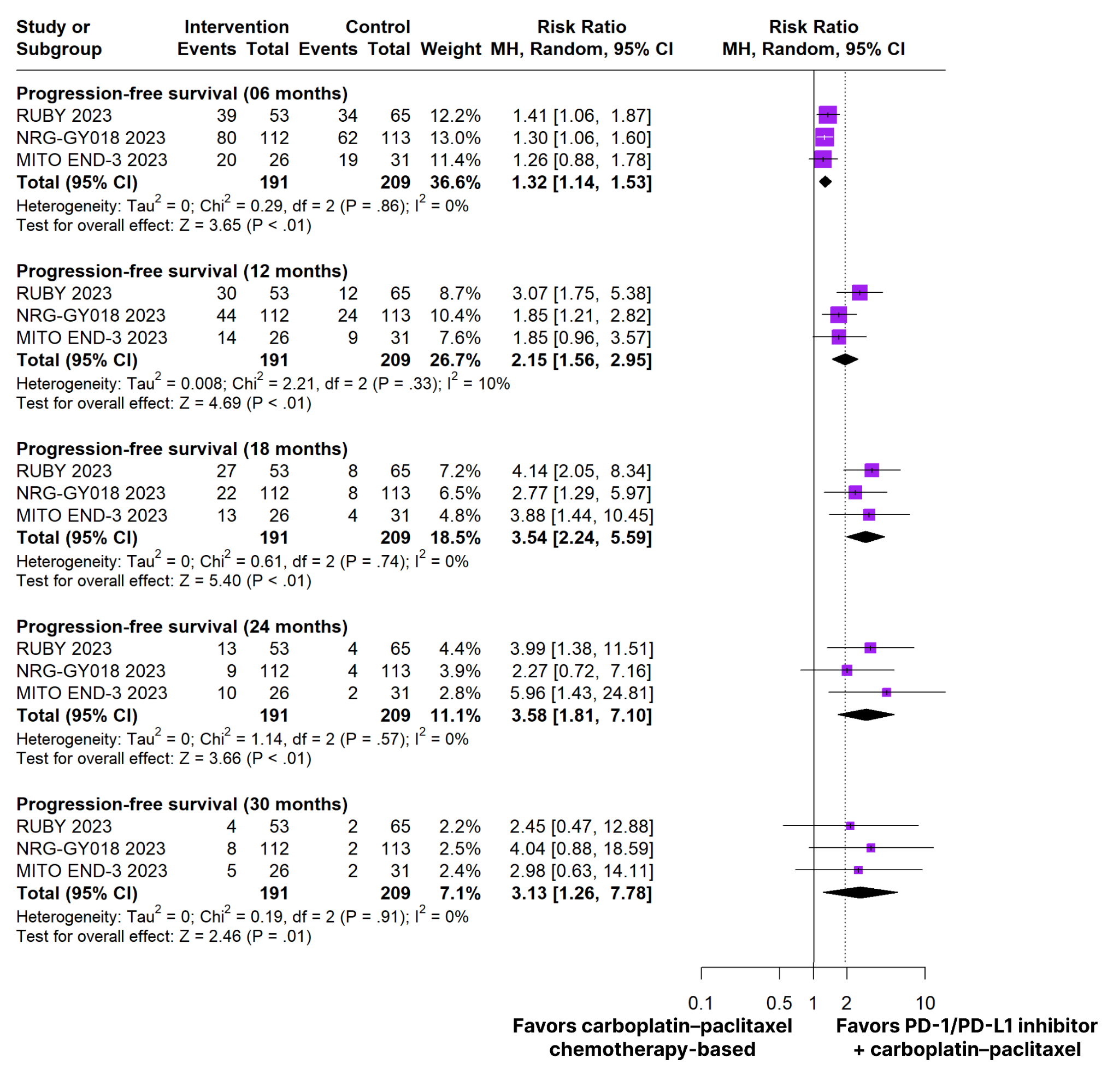


**Figure S1.** Progression-free survival of patients with dMMR (mismatch repair–deficient) endometrial cancer treated with PD-1/PD-L1 inhibitors plus carboplatin and paclitaxel chemotherapy-based versus carboplatin plus paclitaxel chemotherapy-based.


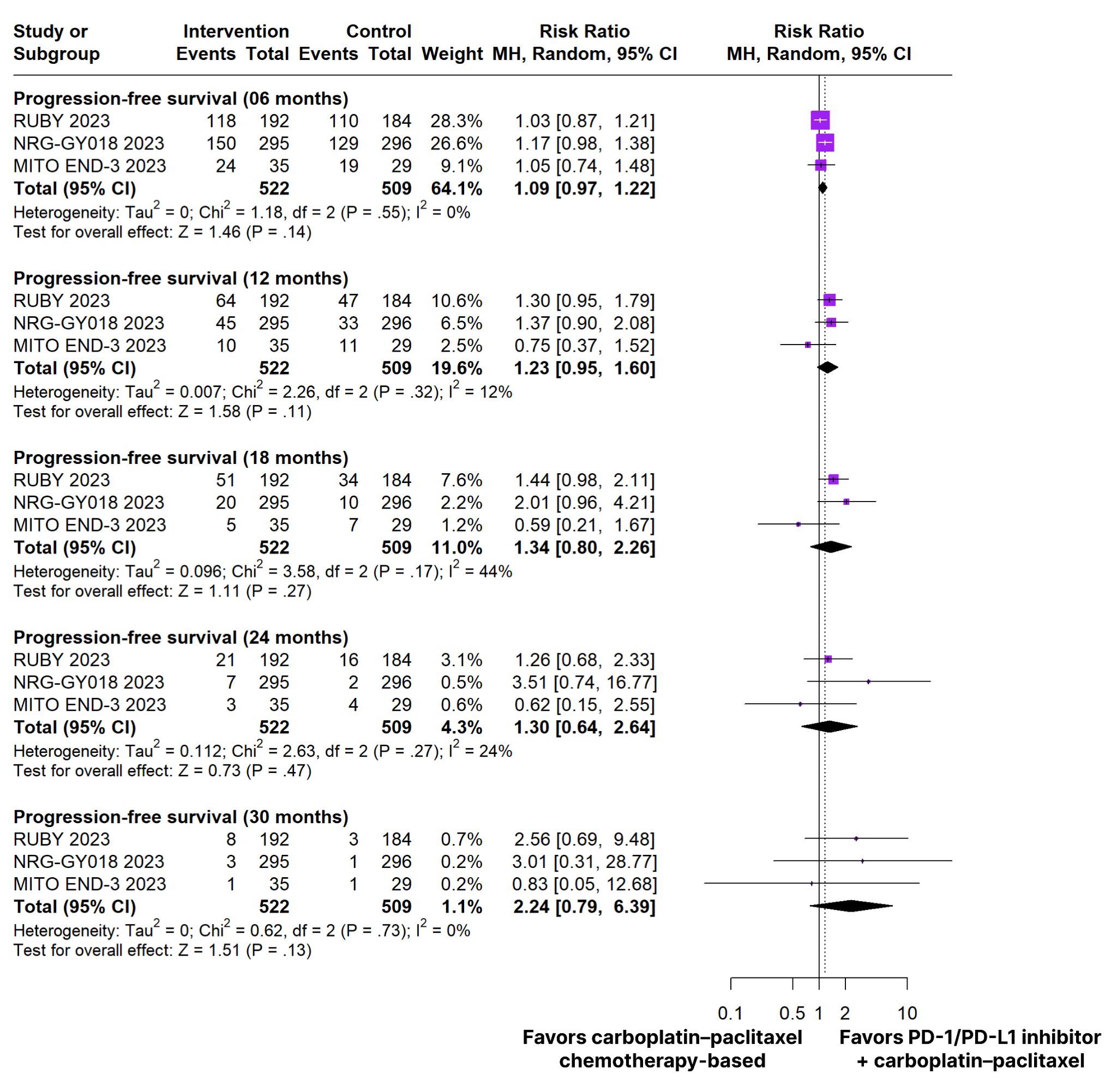


**Figure S2.** Progression-free survival of patients with pMMR (mismatch repair–proficient) endometrial cancer treated with PD-1/PD-L1 inhibitors plus carboplatin and paclitaxel chemotherapy-based versus carboplatin plus paclitaxel chemotherapy-based.

**
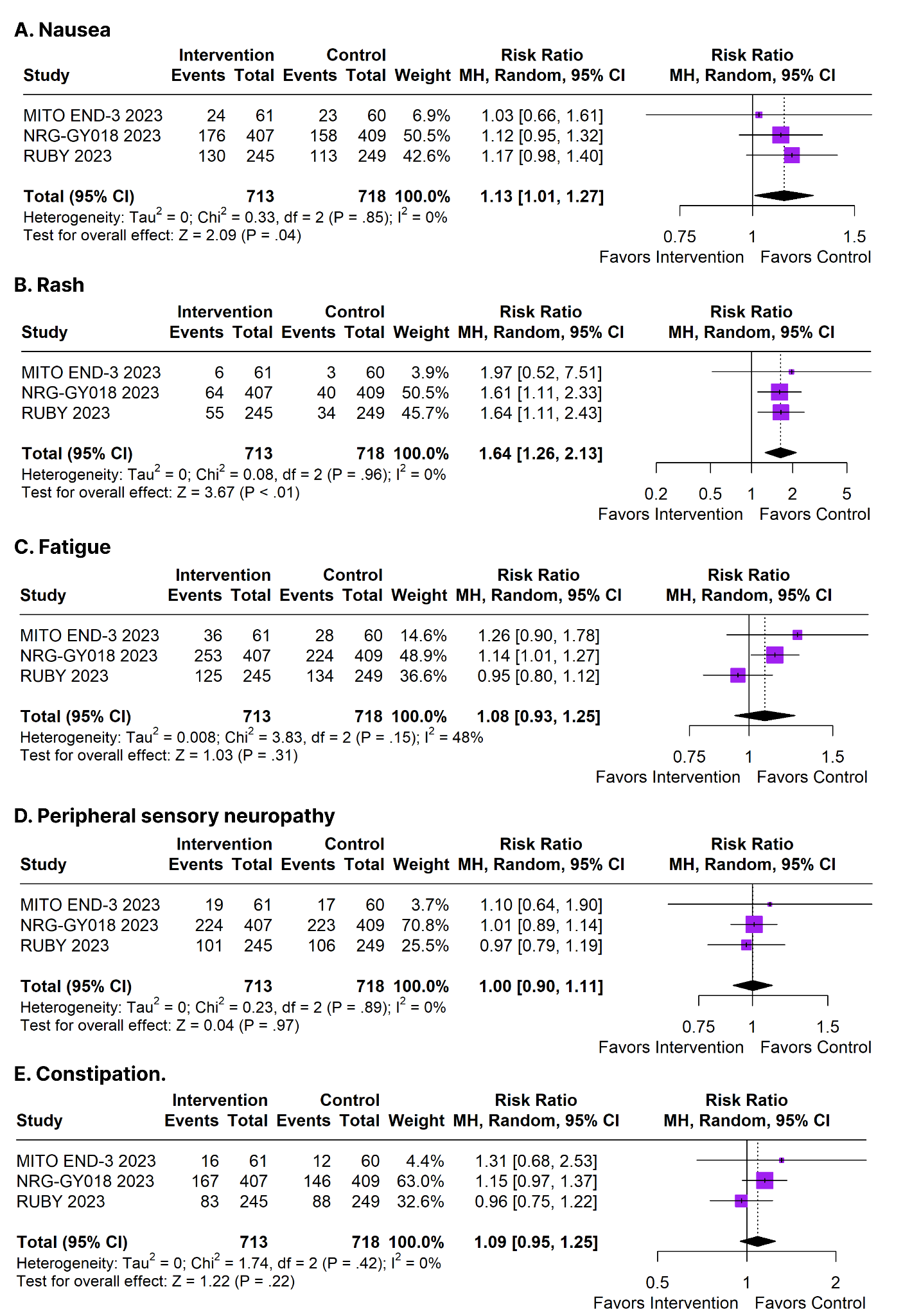
**

**Figure S3.** Any grade of adverse events. **A.** Nausea. **B.** Rash. **C.** Fatigue. **D.** Peripheral sensory neuropathy. **E.** Constipation.


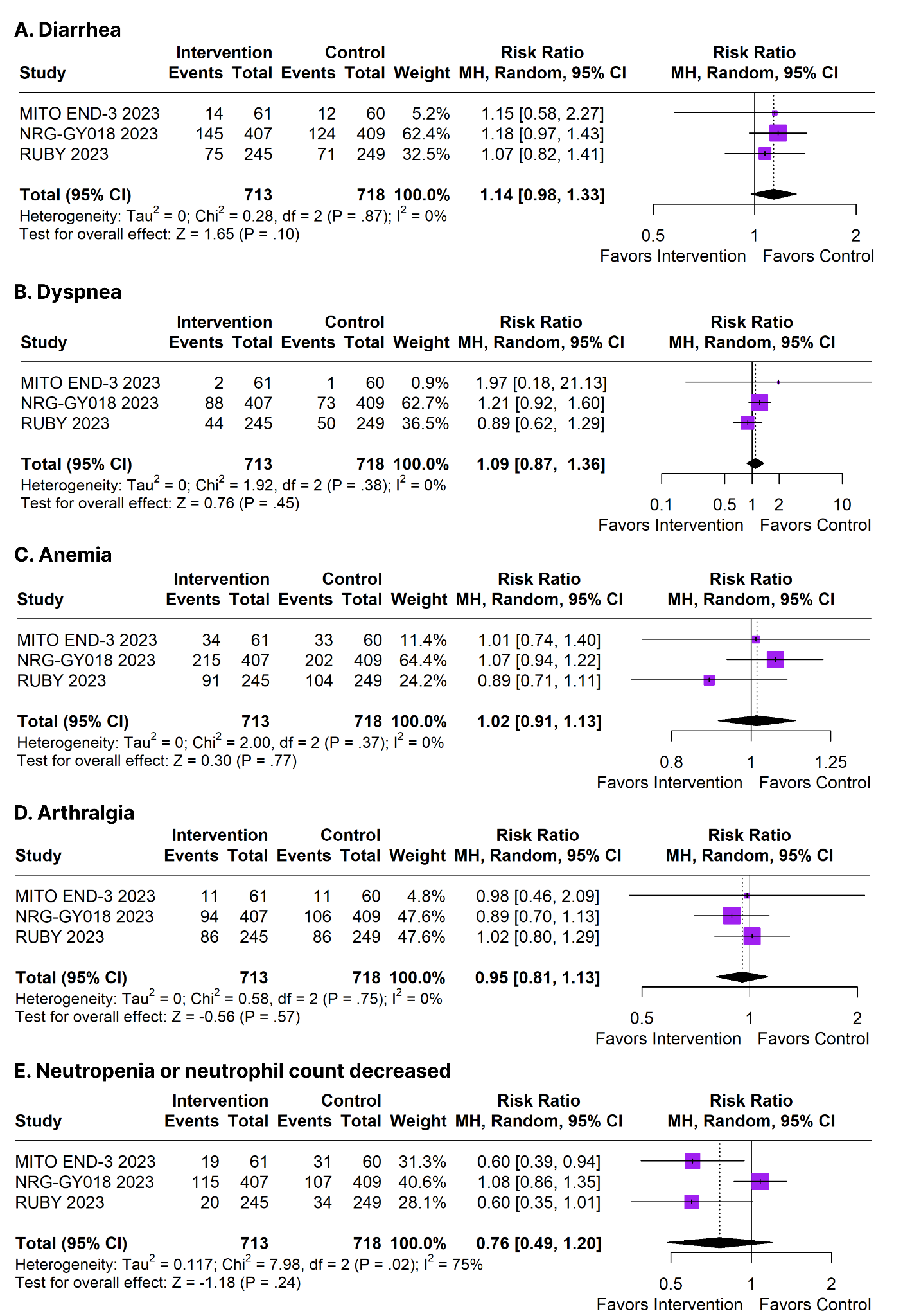


**Figure S4.** Any grade of adverse events. **A.** Diarrhea. **B.** Dyspnea. **C.** Anemia. **D.** Arthralgia. **E.** Neutropenia or neutrophil count decreased.


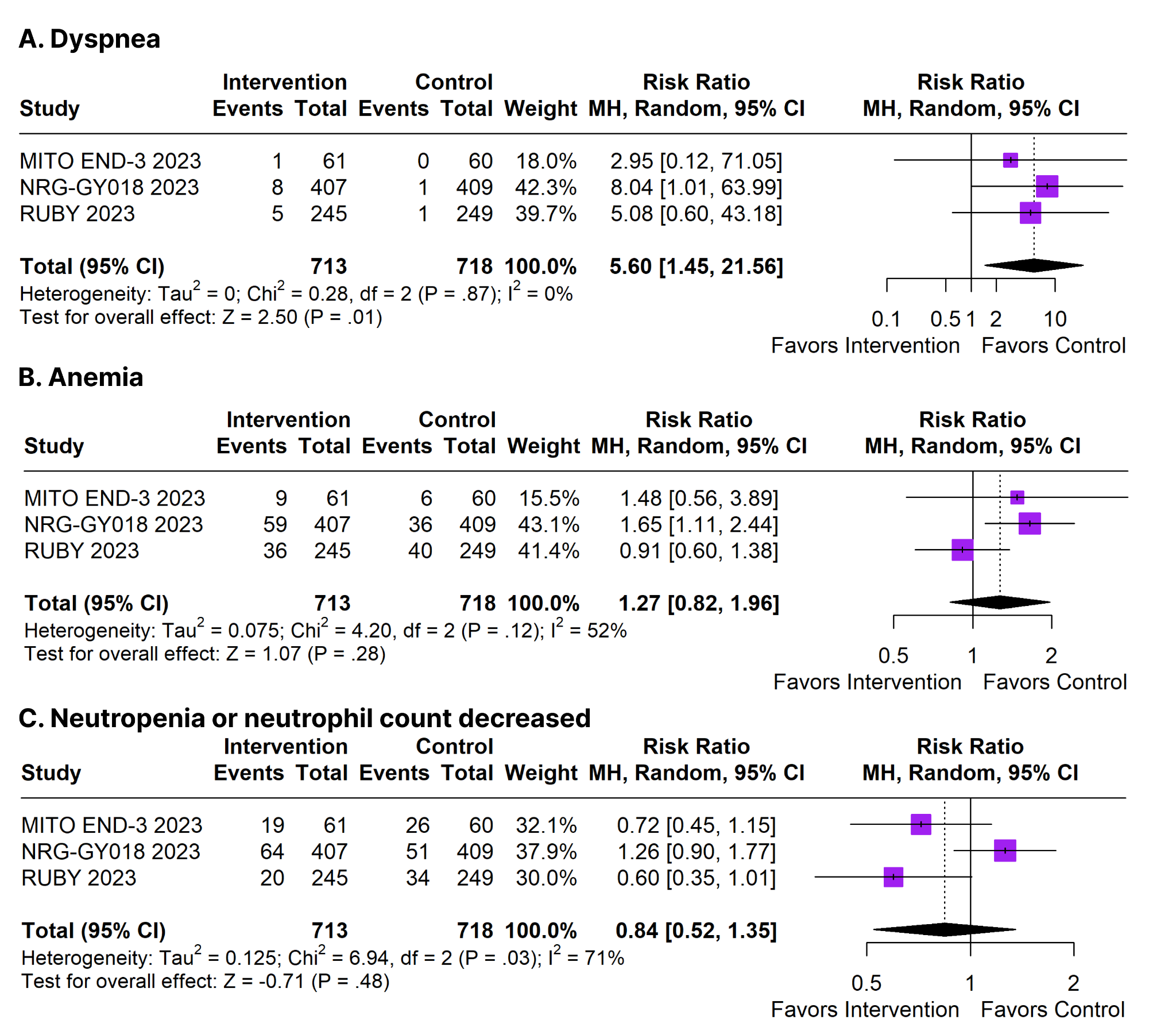


**Figure S5.** Grade ≥3 adverse events **A.** Dyspnea. **B.** Anemia. **C.** Neutropenia or neutrophil count decreased.


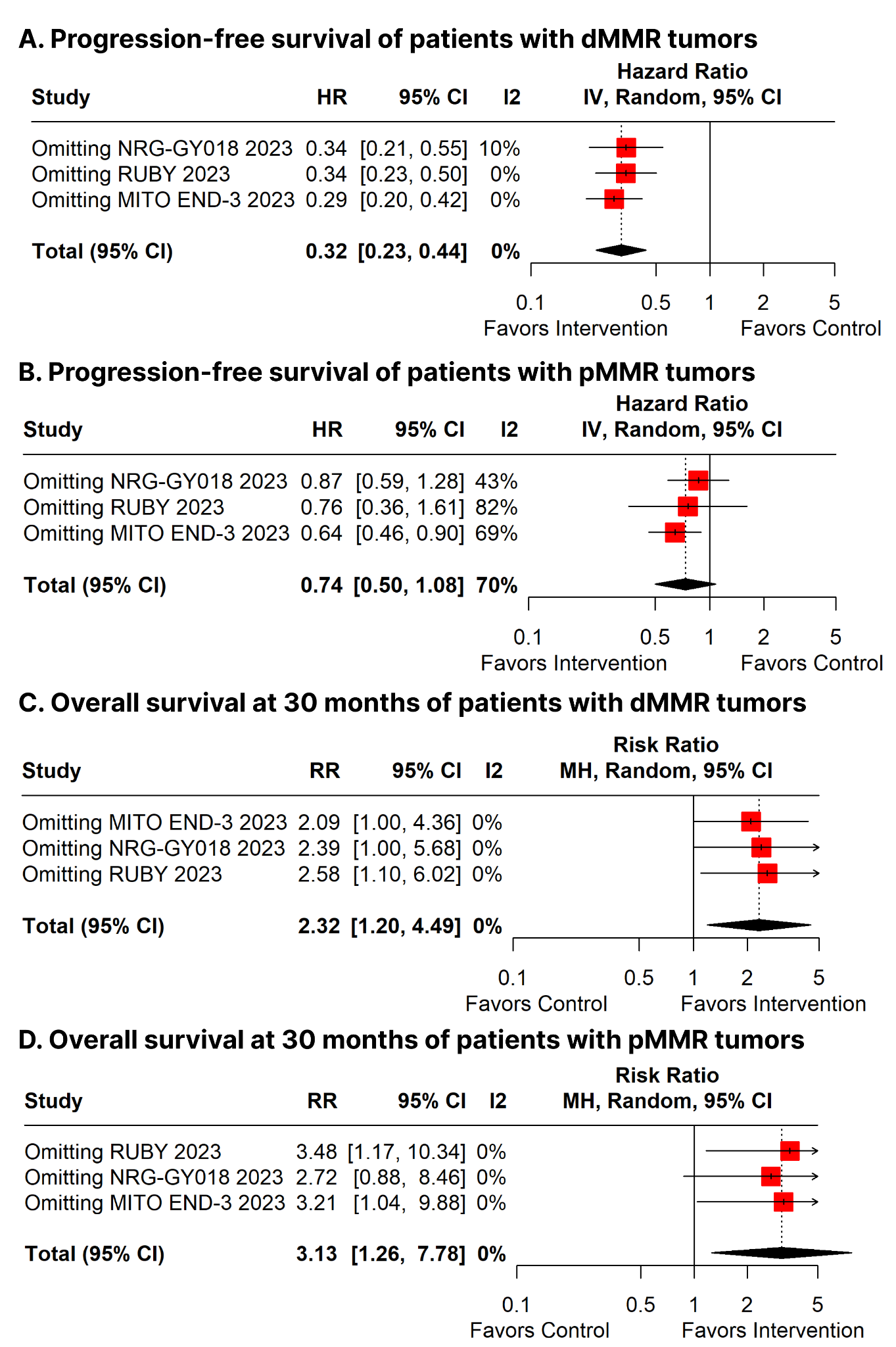


**Figure S6.** Leave-one-out sensitivity analyses. **A.** Progression-free survival of patients with dMMR (mismatch repair–deficient) tumors. **B.** Progression-free survival of patients with pMMR (mismatch repair–proficient) tumors. **C.** Overall survival at 30 months of patients with dMMR (mismatch repair–deficient) tumors. **D.** Overall survival at 30 months of patients with pMMR (mismatch repair–proficient) tumors.
